# TRY – a global database of plant traits

**DOI:** 10.1111/j.1365-2486.2011.02451.x

**Published:** 2011-09

**Authors:** J Kattge, S Díaz, S Lavorel, I C Prentice, P Leadley, G Bönisch, E Garnier, M Westoby, P B Reich, I J Wright, J H C Cornelissen, C Violle, S P Harrison, P M Van Bodegom, M Reichstein, B J Enquist, N A Soudzilovskaia, D D Ackerly, M Anand, O Atkin, M Bahn, T R Baker, D Baldocchi, R Bekker, C C Blanco, B Blonder, W J Bond, R Bradstock, D E Bunker, F Casanoves, J Cavender-Bares, J Q Chambers, F S Chapin, J Chave, D Coomes, W K Cornwell, J M Craine, B H Dobrin, L Duarte, W Durka, J Elser, G Esser, M Estiarte, W F Fagan, J Fang, F Fernández-Méndez, A Fidelis, B Finegan, O Flores, H Ford, D Frank, G T Freschet, N M Fyllas, R V Gallagher, W A Green, A G Gutierrez, T Hickler, S I Higgins, J G Hodgson, A Jalili, S Jansen, C A Joly, A J Kerkhoff, D Kirkup, K Kitajima, M Kleyer, S Klotz, J M H Knops, K Kramer, I Kühn, H Kurokawa, D Laughlin, T D Lee, M Leishman, F Lens, T Lenz, S L Lewis, J Lloyd, J Llusià, F Louault, S Ma, M D Mahecha, P Manning, T Massad, B E Medlyn, J Messier, A T Moles, S C Müller, K Nadrowski, S Naeem, Ü Niinemets, S Nöllert, A Nüske, R Ogaya, J Oleksyn, V G Onipchenko, Y Onoda, J Ordoñez, G Overbeck, W A Ozinga, S Patiño, S Paula, J G Pausas, J Peñuelas, O L Phillips, V Pillar, H Poorter, L Poorter, P Poschlod, A Prinzing, R Proulx, A Rammig, S Reinsch, B Reu, L Sack, B Salgado-Negret, J Sardans, S Shiodera, B Shipley, A Siefert, E Sosinski, J-F Soussana, E Swaine, N Swenson, K Thompson, P Thornton, M Waldram, E Weiher, M White, S White, S J Wright, B Yguel, S Zaehle, A E Zanne, C Wirth

**Affiliations:** * Max-Planck-Institute for Biogeochemistry07745 Jena, Germany; † Instituto Multidisciplinario de Biología Vegetal, Universidad Nacional de Córdoba5000 Córdoba, Argentina; ‡ Laboratoire d'Ecologie Alpine (LECA)CNRS, 38041 Grenoble, France; § Department of Biological Sciences, Macquarie UniversitySydney, NSW 2109, Australia; ¶ Laboratoire d'Ecologie, Systématique et Evolution (ESE), Université Paris-Sud91495 Paris, France; | Centre d'Ecologie Fonctionnelle et Evolutive, CNRS34293 Montpellier, France; ** Department of Forest Resources and Institute of the Environment, University of Minnesota, St. PaulMN 55108, USA; †† Hawkesbury Institute for the Environment, University of Western SydneyRichmond NSW 2753 Australia; ‡‡ Faculty of Earth and Life Sciences, Vrije Universiteit Amsterdam1081 HV Amsterdam, The Netherlands; §§ Department of Ecology and Evolutionary Biology, University of ArizonaTucson, AZ 85721, USA; ¶¶ Department of Integrative Biology, University of CaliforniaBerkeley, CA 94720-3140, USA; || School of Environmental Sciences, University of GuelphOntario, N1G 2W1 Guelph, Canada; *** Research School of Biology, Australian National UniversityCanberra, ACT 0200, Australia; ††† Institute of Ecology, University of Innsbruck6020 Innsbruck, Austria; ‡‡‡ School of Geography, University of LeedsLS2 9JT West Yorkshire, UK; §§§ Department of Environmental Science & Atmospheric Science Center, University of CaliforniaBerkeley, CA 94720, USA; ¶¶¶ Centre for Life Sciences, University of Groningen9700 CC Groningen, The Netherlands; ||| Departamento de Ecologia, Universidade Federal do Rio Grande do Sul91501-970 Porto Alegre, Brasil; **** Department of Botany, University of Cape Town7701 Rondebosch, South Africa; †††† School of Biological Science, University of Wollongong2522 Wollongong, NSW, Australia; ‡‡‡‡ Department of Biological Sciences, New Jersey Institute of TechnologyNewark, NJ 07102, USA; §§§§ Tropical Agricultural Centre for Research and Higher Education (CATIE)93-7170 Turrialba, Costa Rica; ¶¶¶¶ Department of Ecology, Evolution, and Behavior, University of Minnesota, St. PaulMN 55108, USA; |||| Climate Sciences Department, Lawrence Berkeley National LaboratoryBerkeley, CA 94720, USA; ***** Institute of Arctic Biology, University of Alaska FairbanksFairbanks, AK 99775, USA; ††††† Laboratoire Evolution et Diversité Biologique, CNRSToulouse, France; ‡‡‡‡‡ Department of Plant Sciences, University of CambridgeCB3 2EA Cambridge, UK; §§§§§ Division of Biology, Kansas State UniversityKS 66506 Manhattan, USA; ¶¶¶¶¶ Departamento de Ecologia, Federal University of Rio Grande do Sul91540-000 Porto Alegre, Brazil; ||||| Department of Community Ecology, Helmholtz Centre for Environmental Research06120 Halle, Germany; ****** School of Life Sciences, Arizona State UniversityTempe, AZ 85287-4501, USA; †††††† Institute for Plant Ecology, Justus-Liebig-University35392 Giessen, Germany; ‡‡‡‡‡‡ Global Ecology Unit CREAF-CEAB-CSIC, Universitat Autònoma de Barcelona08193 Barcelona, Spain; §§§§§§ Department of Biology, University of Maryland, College ParkMD 20742, USA; ¶¶¶¶¶¶ Department of Ecology, University of Peking100871 Beijing, China; |||||| Departamento de Ciencias Forestales, Universidad del TolimaTolima, Colombia; ******* Department of Ecology, Universidade de São Paulo05508900 São Paulo, Brazil; ††††††† PVBMT, Université de la Réunion97410 Saint Pierre, France; ‡‡‡‡‡‡‡ Department of Biology, University of YorkBath, UK; §§§§§§§ Department of Organismic and Evolutionary Biology, Harvard UniversityMA 02138, USA; ¶¶¶¶¶¶¶ Department of Ecological Modelling, Helmholtz Centre for Environmental Research04318 Leipzig, Germany; ||||||| LOEWE Biodiversity and Climate Research Centre60325 Frankfurt, Germany; ******** Institut für Physische Geographie, Goethe-University Frankfurt60438 Frankfurt, Germany; †††††††† Department of Botany, University of SheffieldSheffield, UK; ‡‡‡‡‡‡‡‡ Department of Botany, Research Institute of Forests and RangelandsTehran, Iran; §§§§§§§§ Institute for Systematic Botany and Ecology, Ulm University89081 Ulm, Germany; ¶¶¶¶¶¶¶¶ Department of Plant Biology, State University of CampinasCP 6109 Campinas, Brazil; |||||||| Departments for Biology and Mathematics, Kenyon CollegeGambier, OH 43022, USA; ********* Herbarium, Library Art and ArchivesThe Royal Botanic Gardens, Kew, TW9 3AE London, UK; ††††††††† Department of Biology, University of FloridaGainesville, FL, USA; ‡‡‡‡‡‡‡‡‡ Institute of Biology and Environmental Sciences, University of Oldenburg26129 Oldenburg, Germany; §§§§§§§§§ School of Biological Sciences, University of NebraskaLincoln, NE 68588-0118, USA; ¶¶¶¶¶¶¶¶¶ Vegetation and Landscape EcologyAlterra, 6700 Wageningen, The Netherlands; ||||||||| Graduate School of Life Sciences, Tohoku University980-8578 Sendai, Japan; ********** School of Forestry, Northern Arizona UniversityFlagstaff, AZ 86011, USA; †††††††††† Department of Biology, University of Wisconsin-Eau ClaireEau Claire, WI 54701, USA; ‡‡‡‡‡‡‡‡‡‡ The Netherlands Centre for Biodiversity Naturalis2300 RA Leiden, The Netherlands; §§§§§§§§§§ James Cook UniversityQld 4870 Cairns, Australia; ¶¶¶¶¶¶¶¶¶¶ Grassland Ecosystem Research, INRA63100 Clermont-Ferrand, France; |||||||||| Department of Environmental Science, University of CaliforniaBerkeley, CA 94720-3140, USA; *********** School of Agriculture, Newcastle UniversityNE1 7RU Newcastle, UK; ††††††††††† School of Biological Earth and Environmental Sciences, University New South Wales2031 Sydney, NSW, Australia; ‡‡‡‡‡‡‡‡‡‡‡ Institute for Special Botany and Functional Biodiversity, University of Leipzig04103 Leipzig, Germany; §§§§§§§§§§§ Department of Ecology, Evolution and Environmental Biology, Columbia UniversityNY, USA; ¶¶¶¶¶¶¶¶¶¶¶ Department of Plant Physiology, Estonian University of Life Sciences51014 Tartu, Estonia; ||||||||||| Institute of Dendrology, Polish Academy of Sciences62-035 Kornik, Poland; ************ Department of Geobotany, Moscow State University119991 Moscow, Russia; †††††††††††† Department Biology, Faculty of Science, Kyushu University812-8581 Fukuoka, Japan; ‡‡‡‡‡‡‡‡‡‡‡‡ Law and Governance Group, Wageningen University6706 KN Wageningen, The Netherlands; §§§§§§§§§§§§ Departamento de Botânica, Universidade Federal do Rio Grande do Sul91501-970 Porto Alegre, Brazil; ¶¶¶¶¶¶¶¶¶¶¶¶ Centre for Ecosystem StudiesAlterra, 6700 Wageningen, The Netherlands; |||||||||||| Centro de Investigaciones sobre Desertificación, Spanish National Research Council46113 Valencia, Spain; ************* Plant SciencesForschungszentrum Jülich, 52428 Jülich, Germany; ††††††††††††† Center for Ecosystem Studies, Wageningen University6700 AA Wageningen, The Netherlands; ‡‡‡‡‡‡‡‡‡‡‡‡‡ Institute of Botany, University of Regensburg93040 Regensburg, Germany; §§§§§§§§§§§§§ Laboratoire Ecobio, Université de Rennes35042 Rennes, France; ¶¶¶¶¶¶¶¶¶¶¶¶¶ Biologie Systémique de la Conservation, Université du QuébecTrois-Rivières, Canada; ||||||||||||| Potsdam Institute for Climate Impact Research14412 Potsdam, Germany; ************** Biosystems Division, Risø National Laboratory for Sustainable Energy4000 Roskilde, Denmark; †††††††††††††† Department of Ecology and Evolutionary Biology, University of CaliforniaLos Angeles, CA 90095, USA; ‡‡‡‡‡‡‡‡‡‡‡‡‡‡ Center for Sustainability Science, Hokkaido University060-080 Sapporo, Japan; §§§§§§§§§§§§§§ Département de Biologie, Université de SherbrookeQuébec Sherbrooke, Canada; ¶¶¶¶¶¶¶¶¶¶¶¶¶¶ Department of Biology, Syracuse UniversityNew York, NY 13244, USA; |||||||||||||| Laboratory of Environmental Planning, Embrapa Temperate Agriculture96010-971 Pelotas, Brazil; *************** Biological Sciences, University of AberdeenAB25 2ZD Aberdeen, Scotland, UK; ††††††††††††††† Department of Plant Biology & Ecology, Michigan State UniversityEast Lansing, MI 48824, USA; ‡‡‡‡‡‡‡‡‡‡‡‡‡‡‡ Department of Animal and Plant Sciences, University of SheffieldS10 2TN Sheffield, UK; §§§§§§§§§§§§§§§ Environmental Sciences Division, Oak Ridge National LaboratoryOak Ridge, TN 37831-6301, USA; ¶¶¶¶¶¶¶¶¶¶¶¶¶¶¶ Department of Geography, Leicester UniversityLE1 7RH Leicester, UK; ||||||||||||||| Department of Watershed Sciences, Utah State UniversityLogan, UT 84322-5210, USA; **************** Smithsonian Tropical Research Institute0843-03092 Balboa, Republic of Panama; †††††††††††††††† Laboratoire Ecobio Université de RennesCNRS, 35042 Rennes, France; ‡‡‡‡‡‡‡‡‡‡‡‡‡‡‡‡ Department of Biology, University of MissouriSt. Louis, MO 63121-4400, USA

**Keywords:** comparative ecology, database, environmental gradient, functional diversity, global analysis, global change, interspecific variation, intraspecific variation, plant attribute, plant functional type, plant trait, vegetation model

## Abstract

Plant traits – the morphological, anatomical, physiological, biochemical and phenological characteristics of plants and their organs – determine how primary producers respond to environmental factors, affect other trophic levels, influence ecosystem processes and services and provide a link from species richness to ecosystem functional diversity. Trait data thus represent the raw material for a wide range of research from evolutionary biology, community and functional ecology to biogeography. Here we present the global database initiative named TRY, which has united a wide range of the plant trait research community worldwide and gained an unprecedented buy-in of trait data: so far 93 trait databases have been contributed. The data repository currently contains almost three million trait entries for 69 000 out of the world's 300 000 plant species, with a focus on 52 groups of traits characterizing the vegetative and regeneration stages of the plant life cycle, including growth, dispersal, establishment and persistence. A first data analysis shows that most plant traits are approximately log-normally distributed, with widely differing ranges of variation across traits. Most trait variation is between species (interspecific), but significant intraspecific variation is also documented, up to 40% of the overall variation. Plant functional types (PFTs), as commonly used in vegetation models, capture a substantial fraction of the observed variation – but for several traits most variation occurs within PFTs, up to 75% of the overall variation. In the context of vegetation models these traits would better be represented by state variables rather than fixed parameter values. The improved availability of plant trait data in the unified global database is expected to support a paradigm shift from species to trait-based ecology, offer new opportunities for synthetic plant trait research and enable a more realistic and empirically grounded representation of terrestrial vegetation in Earth system models.

## Introduction

Plant traits – morphological, anatomical, biochemical, physiological or phenological features measurable at the individual level (Violle *et al.*, 2007) – reflect the outcome of evolutionary and community assembly processes responding to abiotic and biotic environmental constraints (Valladares *et al.*, 2007). Traits and trait syndromes (consistent associations of plant traits) determine how primary producers respond to environmental factors, affect other trophic levels and influence ecosystem processes and services (Aerts & Chapin, 2000; Grime, 2001, 2006; Lavorel & Garnier, 2002; Díaz *et al.*, 2004; Garnier & Navas, 2011). In addition, they provide a link from species richness to functional diversity in ecosystems (Díaz *et al.*, 2007). A focus on traits and trait syndromes therefore provides a promising basis for a more quantitative and predictive ecology and global change science (McGill *et al.*, 2006; Westoby & Wright, 2006).

Plant trait data have been used in studies ranging from comparative plant ecology (Grime, 1974; Givnish, 1988; Peat & Fitter, 1994; Grime *et al.*, 1997) and functional ecology (Grime, 1977; Reich *et al.*, 1997; Wright *et al.*, 2004) to community ecology (Shipley *et al.*, 2006; Kraft *et al.*, 2008), trait evolution (Moles *et al.*, 2005a), phylogeny reconstruction (Lens *et al.*, 2007), metabolic scaling theory (Enquist *et al.*, 2007), palaeobiology (Royer *et al.*, 2007), biogeochemistry (Garnier *et al.*, 2004; Cornwell *et al.*, 2008), disturbance ecology (Wirth, 2005; Paula & Pausas, 2008), plant migration and invasion ecology (Schurr *et al.*, 2005), conservation biology (Ozinga *et al.*, 2009; Römermann *et al.*, 2009) and plant geography (Swenson & Weiser, 2010). Access to trait data for a large number of species allows testing levels of phylogenetic conservatism, a promising principle in ecology and evolutionary biology (Wiens *et al.*, 2010). Plant trait data have been used for the estimation of parameter values in vegetation models, but only in a few cases based on systematic analyses of trait spectra (White *et al.*, 2000; Kattge *et al.*, 2009; Wirth & Lichstein, 2009; Ziehn *et al.*, 2011). Recently, plant trait data have been used for the validation of a global vegetation model as well (Zaehle & Friend, 2010).

While there have been initiatives to compile datasets at regional scale for a range of traits [e.g. LEDA (Life History Traits of the Northwest European Flora: http://www.leda-traitbase.org), BiolFlor (Trait Database of the German Flora: http://www.ufz.de/biolflor), EcoFlora (The Ecological Flora of the British Isles: http://www.ecoflora.co.uk), BROT (Plant Trait Database for Mediterranean Basin Species: http://www.uv.es/jgpausas/brot.htm)] or at global scale focusing on a small number of traits [e.g. GlopNet (Global Plant Trait Network: http://www.bio.mq.edu.au/~iwright/glopian.htm), SID (Seed Information Database: http://data.kew.org/sid/)], a unified initiative to compile data for a large set of relevant plant traits at the global scale was lacking. As a consequence studies on trait variation so far have either been focussed on the local to regional scale including a range of different traits (e.g. Baraloto *et al.*, 2010), while studies at the global scale were restricted to individual aspects of plant functioning, e.g. the leaf economic spectrum (Wright *et al.*, 2004), the evolution of seed mass (Moles *et al.*, 2005a, b) or the characterization of the wood economic spectrum (Chave *et al.*, 2009). Only few analyses on global scale have combined traits from different functional aspects, but for a limited number of plant species (e.g. Díaz *et al.*, 2004).

In 2007, the TRY initiative (TRY – not an acronym, rather an expression of sentiment: http://www.try-db.org) started compiling plant trait data from the different aspects of plant functioning on global scale to make the data available in a consistent format through one single portal. Based on a broad acceptance in the plant trait community (so far 93 trait databases have been contributed, Table 1), TRY has accomplished an unprecedented coverage of trait data and is now working towards a communal global repository for plant trait data. The new database initiative is expected to contribute to a more realistic and empirically based representation of plant functional diversity on global scale supporting the assessment and modelling of climate change impacts on biogeochemical fluxes and terrestrial biodiversity (McMahon *et al.*, 2011).

**Table 1:** Databases currently contributing to the TRY database

Name of the Database		Contact person(s)	Reference(s)
*Databases public, maintained on the Internet*			
1	Seed Information Database (SID)*	J. Dickie, K. Liu	Royal Botanic Gardens Kew Seed Information Database (SID), (2008)
2	Ecological Flora of the British Isles*	A. Fitter, H. Ford	Fitter & Peat (1994)
3	VegClass CBM Global Database	A. Gillison	Gillison & Carpenter (1997)
4	PLANTSdata*	W. A. Green	Green (2009)
5	The LEDA Traitbase*	M. Kleyer	Kleyer *et al.* (2008)
6	BiolFlor Database*	I. Kühn, S. Klotz	Klotz *et al.* (2002), Kühn *et al.* (2004)
7	BROT plant trait database*	J. G. Pausas, S. Paula	Paula & Pausas (2009), Paula *et al.* (2009)
*Databases public, fixed*			
8	Tropical Respiration Database	J. Q. Chambers	Chambers *et al.* (2004, 2009)
9	ArtDeco Database*	W. K. Cornwell, J. H. C. Cornelissen	Cornwell *et al.* (2008)
10	The Americas N&P database	B. J. Enquist, A. J. Kerkhoff	Kerkhoff *et al.* (2006)
11	ECOCRAFT	B. E. Medlyn	Medlyn and Javis (1999), Medlyn *et al.* (1999, 2001)
12	Tree Tolerance Database*	Ü. Niinemets	Niinemets & Valladares (2006)
13	Leaf Biomechanics Database*	Y. Onoda	Onoda *et al.* (2011)
14	BIOPOP: Functional Traits for Nature Conservation*	P. Poschlod	Poschlod *et al.* (2003)
15	BIOME-BGC Parameterization Database*	M. White, P. Thornton	White *et al.* (2000)
16	GLOPNET – Global Plant Trait Network Database*	I. J. Wright, P. B. Reich	Wright *et al.* (2004, 2006)
17	Global Wood Density Database*	A. E. Zanne, J. Chave	Chave *et al.* (2009), Zanne *et al.* (2009)
*Databases not-public, fixed in the majority of cases*			
18	Plant Traits in Pollution Gradients Database	M. Anand	Unpublished data
19	Plant Physiology Database	O. Atkin	Atkin *et al.* (1997, 1999), Loveys *et al.* (2003), Campbell *et al.* (2007)
20	European Mountain Meadows Plant Traits Database	M. Bahn	Bahn *et al.* (1999), Wohlfahrt *et al.* (1999)
21	Photosynthesis Traits Database	D. Baldocchi	Wilson *et al.* (2000), Xu & Baldocchi (2003)
22	Photosynthesis and Leaf Characteristics Database	B. Blonder, B. Enquist	Unpublished data
23	Wetland Dunes Plant Traits Database	P. M. van Bodegom	Bakker *et al.* (2005, 2006), van Bodegom *et al.* (2005, 2008)
24	Ukraine Wetlands Plant Traits Database	P. M. van Bodegom	Unpublished data
25	Plants Categorical Traits Database	P. M. van Bodegom	Unpublished data
26	South African Woody Plants Trait Database (ZLTP)	W. J. Bond, M. Waldram	Unpublished data
27	Australian Fire Ecology Database*	R. Bradstock	Unpublished data
28	Cedar Creek Plant Physiology Database	D. E. Bunker, S. Naeem	Unpublished data
29	Floridian Leaf Traits Database	J. Cavender-Bares	Cavender-Bares et al. (2006)
30	Tundra Plant Traits Databases	F. S. Chapin III	Unpublished data
31	Global Woody N&P Database*	G. Esser, M. Clüsener-Godt	Clüsener-Godt (1989)
32	Abisko & Sheffield Database	J. H. C. Cornelissen	Cornelissen (1996), Cornelissen *et al.* (1996, 1997, 1999, 2001, 2003a, 2004), Castro-Diez *et al.* (1998, 2000), Quested *et al.* (2003)
33	Jasper Ridge Californian Woody Plants Database	W. K. Cornwell, D. D. Ackerly	Cornwell *et al.* (2006), Preston *et al.* (2006), Ackerly & Cornwell (2007), Cornwell & Ackerly (2009)
34	Roots Of the World (ROW) Database	J. M. Craine	Craine *et al.* (2005)
35	Global 15N Database	J. M. Craine	Craine *et al.* (2009)
36	CORDOBASE	S. Díaz	Díaz *et al.* (2004)
37	Sheffield-Iran-Spain Database*	S. Díaz	Díaz *et al.* (2004)
38	Chinese Leaf Traits Database	J. Fang	Han *et al.* (2005), He *et al.* (2006, 2008)
39	Costa Rica Rainforest Trees Database	B. Finegan, B. Salgado	Unpublished data
40	Plant Categorical Traits Database	O. Flores	Unpublished data
41	Subarctic Plant Species Trait Database	G. T. Freschet, J. H. C. Cornelissen	Freschet *et al.* (2010a, b)
42	Climbing Plants Trait Database	R. V. Gallagher	Gallagher *et al.* (2011)
43	The VISTA Plant Trait Database	E. Garnier, S. Lavorel	Garnier *et al.* (2007), Pakeman *et al.* (2008, 2009), Fortunel *et al.* (2009)
44	VirtualForests Trait Database	A. G. Gutiérrez	Gutiérrez (2010)
45	Dispersal Traits Database	S. Higgins	Unpublished data
46	Herbaceous Traits from the Öland Island Database	T. Hickler	Hickler (1999)
47	Global Wood Anatomy Database	S. Jansen, F. Lens	Unpublished data
48	Gobal Leaf Element Composition Database	S. Jansen	Watanabe *et al.* (2007)
49	Leaf Physiology Database*	J. Kattge, C. Wirth	Kattge *et al.* (2009)
50	KEW African Plant Traits Database	D. Kirkup	Kirkup *et al.* (2005)
51	Photosynthesis Traits Database	K. Kramer	Unpublished data
52	Traits of Bornean Trees Database	H. Kurokawa	Kurokawa & Nakashizuka (2008)
53	Ponderosa Pine Forest Database	D. Laughlin	Laughlin *et al.* (2010)
54	New South Wales Plant Traits Database	M. Leishman	Unpublished data
55	The RAINFOR Plant Trait Database	J. Lloyd, N. M. Fyllas	Baker *et al.* (2009), Fyllas *et al.* (2009), Patiño *et al.* (2009)
56	French Grassland Trait Database	F. Louault, J. -F. Soussana	Louault *et al* (2005)
57	The DIRECT Plant Trait Database	P. Manning	Unpublished data
58	Leaf Chemical Defense Database	T. Massad	Unpublished data
59	Panama Leaf Traits Database	J. Messier	Messier *et al.* (2010)
60	Global Seed Mass Database*	A. T. Moles	Moles *et al.* (2004, 2005a, b)
61	Global Plant Height Database*	A. T. Moles	Moles *et al.* (2004)
62	Global Leaf Robustness and Physiology Database	Ü. Niinemets	Niinemets (1999, 2001)
63	The Netherlands Plant Traits Database	J. Ordoñez, P. M. van Bodegom	Ordonez *et al.* (2010a, b)
64	The Netherlands Plant Height Database	W. A. Ozinga	Unpublished data
65	Hawaiian Leaf Traits Database	J. Peñuelas, Ü. Niinemets	Peñuelas *et al.* (2010a, b)
66	Catalonian Mediterranean Forest Trait Database	J. Peñuelas, R. Ogaya	Ogaya & Peñuelas (2003, 2006, 2007, 2008), Sardans *et al.* (2008a, b)
67	Catalonian Mediterranean Shrubland Trait Database	J. Penuelas, M. Estiarte	Peñuelas *et al.* (2007), Prieto *et al.* (2009)
68	ECOQUA South American Plant Traits Database	V. Pillar, S. Müller	Pillar & Sosinski (2003), Overbeck (2005), Blanco *et al.* (2007), Duarte *et al.* (2007), Müller *et al.* (2007), Overbeck & Pfadenhauer (2007)
69	The Tansley Review LMA Database*	H. Poorter	Poorter *et al.* (2009)
70	Categorical Plant Traits Database	H. Poorter	Unpublished data
71	Tropical Rainforest Traits Database	L. Poorter	Poorter & Bongers (2006), Poorter (2009)
72	Frost Hardiness Database*	A. Rammig	Unpublished data
73	Reich-Oleksyn Global Leaf N, P Database	P. B. Reich, J. Oleksyn	Reich *et al.* (2009)
74	Global A, N, P, SLA Database	P. B. Reich	Reich *et al.* (2009)
75	Cedar Creek Savanna SLA, C, N Database	P. B. Reich	Willis *et al.* (2010)
76	Global Respiration Database	P. B. Reich	Reich *et al.* (2008)
77	Leaf and Whole-Plant Traits Database: Hydraulic and Gas Exchange Physiology, Anatomy, Venation Structure, Nutrient Composition, Growth and Biomass Allocation	L. Sack	Sack *et al.* (2003, 2005, 2006), Sack (2004), Nakahashi *et al.* (2005), Sack & Frole (2006), Cavender-Bares *et al.* (2007), Choat *et al.* (2007), Cornwell *et al.* (2007), Martin *et al.* (2007), Coomes *et al.* (2008), Hoof *et al.* (2008), Quero *et al.* (2008), Scoffoni *et al.* (2008), Dunbar-Co *et al.* (2009), Hao *et al.* (2010), Waite & Sack (2010), Markesteijn *et al.* (2011)
78	Tropical Traits from West Java Database	S. Shiodera	Shiodera *et al.* (2008)
79	Leaf And Whole Plant Traits Database	B. Shipley	Shipley (1989, 1995), Shipley and Meziane (2002), Shipley & Parent (1991), McKenna & Shipley (1999), Meziane & Shipley (1999a, b, 2001), Pyankov *et al.* (1999), Shipley & Lechowicz (2000), Shipley & Vu (2002), Vile (2005), Kazakou *et al.* (2006), Vile *et al.* (2006)
80	Herbaceous Leaf Traits Database Old Field New York	A. Siefert	Unpublished data
81	FAPESP Brazil Rain Forest Database	E. Sosinski, C. Joly	Unpublished data
82	Causasus Plant Traits Database	N. A. Soudzilovskaia, V. G. Onipchenko, J. H. C. Cornelissen	Unpublished data
83	Tropical Plant Traits From Borneo Database	E. Swaine	Swaine (2007)
84	Plant Habit Database*	C. Violle, B. H. Dobrin, B. J. Enquist	Unpublished data
85	Midwestern and Southern US Herbaceous Species Trait Database	E. Weiher	Unpublished data
86	The Functional Ecology of Trees (FET) Database – Jena*	C. Wirth, J. Kattge	Wirth & Lichstein (2009)
87	Fonseca/Wright New South Wales Database	I. J. Wright	Fonseca *et al.* (2000), McDonald *et al.* (2003)
88	Neotropic Plant Traits Database	I. J. Wright	Wright *et al.* (2007)
89	Overton/Wright New Zealand Database	I. J. Wright	Unpublished data
90	Categorical Plant Traits Database	I. J. Wright	Unpublished data
91	Panama Plant Traits Database	S. J. Wright	Wright *et al.* (2010)
92	Quercus Leaf C&N Database	B. Yguel	Unpublished data
93	Global Vessel Anatomy Database*	A. E. Zanne, D. Coomes	Unpublished data

Databases are separated whether they are at a final stage or still continuously developed, and whether they are or are not publicly available as an electronic resource in the Internet. Databases that are already integrated databases, pooling a range of original databases (e.g. LEDA, GLOPNET) are highlighted by asterisks (*). Contributions are sorted alphabetically by principal contact person. A database can consist of several datasets (268 individual files have currently been imported to the TRY database). Most of the nonpublic databases contain unpublished besides published data.

For several traits the data coverage in the TRY database is sufficient to quantify the relative amount of intra- and interspecific variation, as well as variation within and between different functional groups. Thus, the dataset allows to examine two basic tenets of comparative ecology and vegetation modelling, which, due to lack of data, had not been quantified so far:

1. On the global scale, the aggregation of plant trait data at the species level captures the majority of trait variation. This central assumption of plant comparative ecology implies that, while there is variation within species, this variation is smaller than the differences between species (Garnier *et al.*, 2001; Keddy *et al.*, 2002; Westoby *et al.*, 2002; Shipley, 2007). This is the basic assumption for using average trait values of species to calculate indices of functional diversity (Petchey & Gaston, 2006; de Bello *et al.*, 2010; Schleuter *et al.*, 2010), to identify ecologically important dimensions of trait variation (Westoby, 1998) or to determine the spatial variation of plant traits (Swenson & Enquist, 2007; Swenson & Weiser, 2010).
2. On the global scale, basic plant functional classifications capture a sufficiently important fraction of trait variation to represent functional diversity. This assumption is implicit in today's dynamic global vegetation models (DGVMs), used to assess the response of ecosystem processes and composition to CO_2_ and climate changes. Owing to computational constraints and lack of detailed information these models have been developed to represent the functional diversity of >300 000 documented plant species on Earth with a small number (5–20) of basic plant functional types (PFTs, e.g. Woodward & Cramer, 1996; Sitch *et al.*, 2003). This approach has been successful so far, but limits are becoming obvious and challenge the use of such models in a prognostic mode, e.g. in the context of Earth system models (Lavorel *et al.*, 2008; McMahon *et al.*, 2011).

This article first introduces the TRY initiative and presents a summary of data coverage with respect to different traits and regions. For a range of traits, we characterize general statistical properties of the trait density distributions, a prerequisite for statistical analyses, and provide mean values and ranges of variation. For 10 traits that are central to leading dimensions of plant strategy, we then quantify trait variation with respect to species and PFT and thus examine the two tenets mentioned above. Finally, we demonstrate how trait variation within PFT is currently represented in the context of global vegetation models.

## Material and methods

### Types of data compiled

The TRY data compilation focuses on 52 groups of traits characterizing the vegetative and regeneration stages of plant life cycle, including growth, reproduction, dispersal, establishment and persistence (Table 2). These groups of traits were collectively agreed to be the most relevant for plant life-history strategies, vegetation modelling and global change responses on the basis of existing shortlists (Grime *et al.*, 1997; Weiher *et al.*, 1999; Lavorel & Garnier, 2002; Cornelissen *et al.*, 2003b; Díaz *et al.*, 2004; Kleyer *et al.*, 2008) and wide consultation with vegetation modellers and plant ecologists. They include plant traits *sensu stricto*, but also ‘performances’ (*sensu* Violle *et al.*, 2007), such as drought tolerance or phenology.

**Table 2:** Summary of data coverage in the TRY data repository (March 31, 2011) for the 52 groups of focus traits and one group lumping all other traits (53)

Group of traits		Traits per group	Datasets	Species	Entries	Geo-referenced	Location	Soil
**1**	Plant growth form*	**7**	**62**	**39 715**	**130 527**	**45 683**	**48 355**	**19 630**
2	Plant life form*	1	9	7870	64 949	55 476	58 575	53 008
3	Plant resprouting capacity*	4	7	3248	5219	410	319	2462
4	Plant height	15	63	18 071	105 422	43 351	50 154	34 325
5	Plant longevity	4	23	8198	18 844	3709	2336	5109
6	Plant age of reproductive maturity	3	3	1506	2024	0	24	0
7	Plant architectural relationships	72	43	10 227	356 188	340 540	340 390	332 608
8	Plant crown size	4	8	276	4180	1450	846	33
9	Plant surface roughness	1	1	31	31	0	0	0
10	Plant tolerance to stress	40	14	8275	62 362	877	1286	33 799
11	Plant phenology	10	16	7630	26 765	2900	8816	6868
**12**	Leaf type*	**1**	**15**	**33 519**	**49 668**	**6261**	**4490**	**2511**
13	Leaf compoundness*	1	15	34 523	50 502	13 495	13 558	230
**14**	Leaf photosynthetic pathway*	**1**	**29**	**31 641**	**40 807**	**6305**	**4442**	**5495**
**15**	Leaf phenology type*	**1**	**35**	**15 512**	**65 536**	**36 579**	**37 888**	**24 900**
16	Leaf size	17	67	16 877	205 165	158 066	138 105	74 424
17	Leaf longevity	4	18	1080	1953	1705	1515	551
18	Leaf angle	2	6	4693	41 882	41 848	41 805	39 820
19	Leaf number per unit shoot length	1	4	4135	10 751	1340	2007	1265
20	Leaf anatomy	41	10	1076	26 649	24 014	23 950	0
21	Leaf cell size	14	6	310	1196	339	462	0
22	Leaf mechanical resistance	7	17	4206	11 645	5608	6295	227
23	Leaf absorbance	1	4	137	363	0	0	61
24	Specific leaf area (SLA)	13	89	8751	87 064	63 730	53 830	18 149
25	Leaf dry matter content	5	35	3098	33 777	26 125	19 767	6919
26	Leaf carbon content	3	32	3028	18 887	15 295	11 938	7857
27	Leaf nitrogen content	4	62	7122	58 064	43 417	41 844	25 857
28	Leaf phosphorus content	2	35	4870	26 065	19 022	21 095	7390
29	Tissue carbon content (other plant organs)	19	18	659	4273	2726	2040	1093
30	Tissue nitrogen content (other plant organs)	55	40	4848	32 438	24 598	22 317	21 904
31	Tissue phosphorus content (other plant organs)	16	18	3763	17 058	10 115	12 519	2445
32	Tissue chemical composition (apart from C,N,P)	136	28	5031	84 743	26 272	74 076	25 152
33	Photosynthesis	49	34	2049	19 793	9446	9980	11 127
34	Stomatal conductance	76	23	918	11 811	4386	6409	4729
35	Respiration	105	18	633	14 898	6423	12 519	3621
36	Litter decomposability	2	8	972	2172	2013	1568	968
37	Pollination mode*	1	10	4211	16 571	780	853	299
38	Dispersal mode*	6	19	9728	43 502	5410	6357	341
39	Seed germination stimulation*	6	7	3407	7074	112	206	4437
40	Seed size	17	30	26 839	158 881	13 225	6780	3755
41	Seed longevity	3	5	1862	11 466	3	97	3
42	Seed morphology	5	9	2326	3811	567	1253	0
43	Stem bark thickness	1	3	52	183	183	183	0
44	Wood porosity*	1	1	5221	7059	0	0	0
**45**	Woodiness*	**1**	**23**	**44 385**	**74 891**	**24 957**	**26 237**	**19 609**
46	Wood anatomy	77	13	8506	252 072	126	24	965
47	Wood density	10	34	11 907	43 871	19 422	31 522	3121
48	Modifications for storage*	4	7	4090	10 410	4052	4054	3747
49	Mycorrhiza type*	1	5	2453	14 935	10 481	10 500	10 481
50	Nitrogen fixation capacity*	3	22	10 642	36 023	18 663	16 826	17 627
51	Rooting depth	1	5	613	629	451	453	280
52	Defence/allelopathy/palatability	15	12	3333	13 388	2489	2663	10 936
	Additional traits	257	132	35 286	496 383	123 068	135 052	179 577
	Sum	1146	268 (total)	69 296 (total)	2 884 820	1 267 513	1 318 580	1 029 715

* Qualitative traits assumed to have low variability within species.

Traits that address one plant characteristic but expressed differently are summarized in groups, e.g. the group ‘leaf nitrogen content’ consists of the three traits: leaf nitrogen content per dry mass, leaf nitrogen content per area and nitrogen content per leaf. In the case of respiration, the database contains 105 related traits: different organs, different reference values (e.g. dry mass, area, volume, nitrogen) or characterizing the temperature dependence of respiration (e.g. *Q*_10_). Specific information for each trait is available on the TRY website (http://www.try-db.org). Datasets: number of contributed datasets; Species: number of species characterised by at least one trait entry; Entries: number of trait entries; Georeferenced, Location, Soil: number of trait entries geo-referenced by coordinates, resp. with information about location or soil.

Bold: qualitative traits standardized and made publicly available on the TRY website.

Quantitative traits vary within species as a consequence of genetic variation (among genotypes within a population/species) and phenotypic plasticity. Ancillary information is necessary to understand and quantify this variation. The TRY dataset contains information about the location (e.g. geographical coordinates, soil characteristics), environmental conditions during plant growth (e.g. climate of natural environment or experimental treatment), and information about measurement methods and conditions (e.g. temperature during respiration or photosynthesis measurements). Ancillary data also include primary references.

By preference individual measurements are compiled in the database, like single respiration measurements or the wood density of a specific individual tree. The dataset therefore includes multiple measurements for the same trait, species and site. For some traits, e.g. leaf longevity, such data are only rarely available on single individuals (e.g. Reich *et al.*, 2004), and data are expressed per species per site instead. Different measurements on the same plant (resp. organ) are linked to form observations that are hierarchically nested. The database structure ensures that (1) the direct relationship between traits and ancillary data and between different traits that have been measured on the same plant (resp. organ) is maintained and (2) conditions (e.g. at the stand level) can be associated with the individual measurements (Kattge *et al.*, 2010). The structure is consistent with the Extensible Observation Ontology (OBOE; Madin *et al.*, 2008), which has been proposed as a general basis for the integration of different data streams in ecology.

The TRY dataset combines several preexisting databases based on a wide range of primary data sources, which include trait data from plants grown in natural environments and under experimental conditions, obtained by a range of scientists with different methods. Trait variation in the TRY dataset therefore reflects natural and potential variation on the basis of individual measurements at the level of single organs, and variation due to different measurement methods and measurement error (random and bias).

### Data treatment in the context of the TRY database

**Fig. 1:**
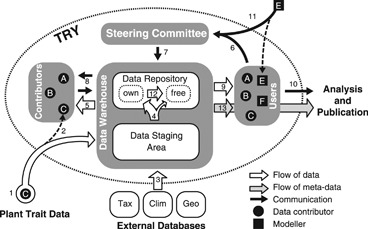
The TRY process of data sharing. Researcher C contributes plant trait data to TRY (1) and becomes a member of the TRY consortium (2). The data are transferred to the Staging Area, where they are extracted and imported, dimensionally and taxonomically cleaned, checked for consistency against all other similar trait entries and complemented with covariates from external databases [3; Tax, taxonomic databases, IPNI/TROPICOS accessed via TaxonScrubber (Boyle, 2006); Clim, climate databases, e.g. CRU; Geo, geographic databases]. Cleaned and complemented data are transferred to the Data Repository (4). If researcher C wants to retain full ownership, the data are labelled accordingly. Otherwise they obtain the status ‘freely available within TRY’. Researcher C can request her/his own data – now cleaned and complemented – at any time (5). If she/he has contributed a minimum amount of data (currently >500 entries), she/he automatically is entitled to request data other than her/his own from TRY. In order to receive data she/he has to submit a short proposal explaining the project rationale and the data requirements to the TRY steering committee (6). Upon acceptance (7) the proposal is published on the Intranet of the TRY website (title on the public domain) and the data management automatically identifies the potential data contributors affected by the request. Researcher C then contacts the contributors who have to grant permission to use the data and to indicate whether they request coauthorship in turn (8). All this is handled via standard e-mails and forms. The permitted data are then provided to researcher C (9), who is entitled to carry out and publish the data analysis (10). To make trait data also available to vegetation modellers – one of the pioneering motivations of the TRY initiative – modellers (e.g. modeller E) are also allowed to directly submit proposals (11) without prior data submission provided the data are to be used for model parameter estimation and evaluation only. We encourage contributors to change the status of their data from ‘own’ to ‘free’ (12) as they have successfully contributed to publications. With consent of contributors this part of the database is being made publicly available without restriction. So far look-up tables for several qualitative traits (see Table 2) have been published on the website of the TRY initiative (http://www.try-db.org). Meta-data are also provided without restriction (13).

The TRY database has been developed as a Data Warehouse (Fig. 1) to combine data from different sources and make them available for analyses in a consistent format (Kattge *et al.*, 2010). The Data Warehouse provides routines for data extraction, import, cleaning and export. Original species names are complemented by taxonomically accepted names, based on a checklist developed by IPNI (The International Plant Names Index: http://www.ipni.org) and TROPICOS (Missouri Botanical Garden: http://www.tropicos.org), which had been made publicly available on the TaxonScrubber website by the SALVIAS (Synthesis and Analysis of Local Vegetation Inventories Across Sites: http://www.salvias.net) initiative (Boyle, 2006). Trait entries and ancillary data are standardized and errors are corrected after consent from data contributors. Finally, outliers and duplicate trait entries are identified and marked (for method of outlier detection, see Appendix S1). The cleaned and complemented data are moved to the data repository, whence they are released on request.

### Selection of data and statistical methods in the context of this analysis

For the analyses in the context of this manuscript, we have chosen traits with sufficient coverage from different aspects of plant functioning. The data were standardized, checked for errors and duplicates excluded. Maximum photosynthetic rates and stomatal conductance were filtered for temperature (15–30 °C), light (PAR >500 μmol m^2^ s^−1^) and atmospheric CO_2_ concentration during measurements (300–400 ppm); data for respiration were filtered for temperature (15–30 °C). A temperature range for respiration from 15–30 °C will add variability to trait values. Nevertheless, an immediate response of respiration to temperature is balanced by an opposite adaptation of basal respiration rates to long-term temperature changes. More detailed analyses will have to take short- and long-term impact of temperature on both scales into account. With respect to photosynthetic rates the problem is similar, but less severe. Statistical properties of density distributions of trait data were characterized by skewness and kurtosis on the original scale and after log-transformation. The Jarque–Bera test was applied to assess departure from normality (Bera & Jarque, 1980). Finally outliers were identified (see supporting information, Appendix S1). The subsequent analyses are based on standardized trait values, excluding outliers and duplicates.

PFTs were defined similar to those used in global vegetation models (e.g. Woodward & Cramer, 1996; Sitch *et al.*, 2003; see Table 5), based on standardized tables for the qualitative traits ‘plant growth form’ (grass, herb, climber, shrub, tree), ‘leaf type’ (needle-leaved, broad-leaved), ‘leaf phenology type’ (deciduous, evergreen), ‘photosynthetic pathway’ (C3, C4, CAM) and ‘woodiness’ (woody, nonwoody).

**Table 5:** Variation within and between species and within and between plant functional types (PFT)

	Seed mass			Plant height			LL			SLA			*N*_m_			*P*_m_			*N*_a_			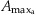			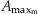			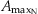		
										
	*n*	Mean	SD	*n*	Mean	SD	*n*	Mean	SD	*n*	Mean	SD	*n*	Mean	SD	*n*	Mean	SD	*n*	Mean	SD	*n*	Mean	SD	*n*	Mean	SD	*n*	Mean	SD
All data	49 837	2.38	1.08	26 624	1.84	0.78	1540	9.40	0.41	45 733	16.60	0.26	33 880	17.40	0.18	17 056	1.23	0.24	12 860	1.59	0.19	3145	10.11	0.25	2919	0.12	0.33	3074	6.23	0.28
*PFT summary*																														
Mean		5.27	0.79		2.67	0.43		11.42	0.25		15.08	0.20		17.46	0.16		1.24	0.21		1.53	0.17		10.22	0.22		0.10	0.24		5.72	0.23
SD between		0.90			0.69			0.40			0.18			0.10			0.14			0.11			0.16			0.24			0.27	
*n*/PFT	2623			1401			91			2407			1783			898			677			208			198			194		
Sign. *P*	***			***			***			***			***			***			***			***			***			***		
*Species summary*																														
Mean		2.12	0.13		3.06	0.18		9.09	0.03		18.84	0.09		18.37	0.08		1.22	0.11		1.48	0.10		10.13	0.14		0.12	0.14		5.79	0.14
SD between		1.03			0.81			0.40			0.22			0.16			0.23			0.16			0.22			0.33			0.25	
nsp	2707			882			363			2423			1250			649			519			168			120			121		
*n*/sp	11			10			3			16			18			16			15			13			11			13		
Sign. *P*	***			***			***			***			***			***			***			***			***			***		
*Plant functional types*																														
Fern (218)	3	0.08	0.83	329	0.75	0.47	13	28.48	0.25	647	18.86	0.22	143	14.77	0.19	91	0.72	0.21	50	1.14	0.20	2	9.15	0.18	2	0.09	0.12	4	1.77	0.39
Grass C3 (594)	3935	0.61	0.70	1242	0.44	0.31	81	3.85	0.22	5033	20.12	0.20	2669	17.84	0.16	1435	1.43	0.23	1075	1.14	0.17	341	13.25	0.21	232	0.20	0.24	215	9.25	0.27
Grass C4 (248)	635	0.58	0.60	383	0.64	0.33	6	1.68	0.18	583	19.23	0.22	1128	14.14	0.15	150	1.36	0.23	232	0.93	0.16	97	19.78	0.20	70	0.25	0.17	80	18.81	0.22
Herb C3 (3129)	15 506	0.77	0.82	3404	0.38	0.38	215	3.49	0.25	18 830	22.83	0.19	4893	23.31	0.16	1870	2.02	0.21	2798	1.29	0.18	1015	12.81	0.25	663	0.21	0.26	694	8.49	0.20
Herb C4 (63)	183	0.49	0.53	36	0.25	0.55		1.00	0.00	212	20.20	0.25	87	18.78	0.24	47	1.86	0.25	127	1.31	0.14	102	21.87	0.22	33	0.15	0.29	89	15.42	0.24
Climber nonwoody (233)	751	15.25	0.57	268	1.05	0.48	17	8.99	0.35	949	23.40	0.20	295	25.34	0.17	143	1.38	0.26	154	1.33	0.19	29	10.04	0.24	30	0.12	0.39	26	5.74	0.28
Climber woody (73)	102	15.16	0.43	76	3.74	0.51	7	16.68	0.35	443	14.73	0.19	157	21.34	0.14	101	1.62	0.23	42	1.32	0.20	13	11.21	0.21	3	0.09	0.20	3	4.10	0.19
Shrub broadleaved deciduous (596)	1573	6.67	0.99	1221	3.59	0.49	167	4.68	0.19	3838	15.36	0.18	2223	21.50	0.14	1209	1.56	0.20	748	1.45	0.18	233	9.97	0.17	242	0.15	0.23	228	6.02	0.18
Shrub broadleaved evergreen (1162)	1911	4.02	0.98	1694	1.61	0.55	284	15.88	0.26	3216	8.99	0.21	2623	13.73	0.18	1504	0.84	0.25	1033	1.90	0.19	390	8.96	0.23	345	0.08	0.29	382	4.57	0.23
Shrub needleleaved (83)	256	2.55	1.28	121	3.53	0.58	17	36.66	0.25	303	7.43	0.15	223	10.11	0.15	123	0.74	0.26	89	1.83	0.17	19	8.03	0.24	19	0.04	0.16	17	4.02	0.25
Tree broadleaved deciduous (699)	1606	33.80	1.09	1471	20.82	0.28	240	5.83	0.17	3963	15.40	0.17	4343	21.32	0.13	2225	1.44	0.20	1723	1.57	0.16	539	9.34	0.18	520	0.12	0.23	360	6.28	0.17
Tree broadleaved evergreen (2136)	1487	27.64	1.07	1973	16.56	0.36	360	16.83	0.29	3859	9.46	0.19	5921	16.89	0.16	3177	0.86	0.20	2723	1.87	0.15	652	7.79	0.23	484	0.07	0.27	564	4.63	0.22
Tree needleleaved deciduous (16)	64	6.88	0.57	88	32.98	0.20	12	6.08	0.01	129	10.09	0.09	248	19.37	0.10	155	1.83	0.15	37	1.80	0.13	11	6.90	0.20	12	0.06	0.18	13	4.17	0.17
Tree needleleaved evergreen (134)	889	13.77	0.63	882	27.20	0.30	63	39.71	0.21	1517	5.00	0.13	5558	12.09	0.10	3622	1.23	0.16	984	2.62	0.14	196	9.45	0.24	121	0.05	0.26	124	3.14	0.25
*Plant species (exemplary)*																														
*Carex bigelowii*	23	0.47	0.304	6	0.23	0.137	2	3.62	0.003	14	12.19	0.124	41	20.32	0.107	16	1.94	0.186	7	1.65	0.059	3	15.16	0.107	3	0.17	0.003	3	8.97	0.059
*Dactylis glomerata*	88	0.81	0.154	39	0.73	0.153	3	2.75	0.125	139	24.58	0.109	50	24.67	0.128	22	1.98	0.183	11	1.32	0.098	7	13.45	0.160	7	0.31	0.194	7	9.82	0.189
*Poa pratensis*	57	0.26	0.139	22	0.50	0.140	1	3.01		169	23.96	0.131	63	17.36	0.172	11	2.28	0.178	6	1.19	0.184	8	13.75	0.200	6	0.17	0.187	8	10.10	0.170
*Trifolium pratense*	61	1.53	0.117	45	0.39	0.277				141	22.85	0.084	34	38.65	0.086	14	2.07	0.123	7	1.65	0.090	5	16.94	0.061	4	0.43	0.116	3	10.99	0.113
*Prunus spinosa*	22	165.01	0.244	14	2.92	0.216	3	5.60	0.024	86	14.54	0.091	16	28.05	0.114	13	2.15	0.099	11	1.87	0.081	3	11.17	0.048	3	0.13	0.074	3	6.32	0.101
*Acacia doratoxylon*	3	15.40	0.000	7	6.09	0.268	3	19.80	0.003	3	4.57	0.000	7	20.37	0.012	6	0.83	0.003	3	4.38	0.001	2	14.51	0.002	2	0.07	0.003	2	3.34	0.001
*Phyllota phylicoides*	6	2.83	0.026	6	0.67	0.345	2	22.43	0.001	6	7.44	0.059	5	12.94	0.025				2	1.49	0.002	2	8.35	0.003	2	0.05	0.003	2	4.87	0.001
*Pultenaea daphnoides*	5	3.98	0.141	3	2.86	0.036	2	9.36	0.002	3	13.76	0.192	6	19.40	0.004	5	0.35	0.013	3	1.83	0.003	2	9.58	0.002	2	0.10	0.001	2	5.06	0.001
*Lepechinia calycina*	4	12.35	0.186	2	2.79	0.174	2	4.39	0.003	5	11.23	0.075	5	18.38	0.139	3	1.20	0.000	3	1.48	0.153	2	12.56	0.001	2	0.13	0.001	2	6.91	0.001
*Leptospermum polygalifolium*	4	0.18	0.056	3	4.00	0.000	2	7.38	0.003	2	10.93	0.002	6	13.35	0.014	5	0.49	0.048	3	1.20	0.001	3	8.56	0.002	2	0.11	0.000	3	7.62	0.024
*Banksia marginata*	7	8.51	0.073	3	5.45	0.326	3	36.36	0.001	11	5.72	0.072	11	8.30	0.050	4	0.34	0.051	8	1.41	0.032	2	19.52	0.001	2	0.10	0.001	2	12.76	0.000
*Grevillea buxifolia*	7	46.39	0.114	6	1.35	0.271	2	15.07	0.003	4	8.18	0.094	6	7.16	0.006	2	0.29	0.000	3	0.78	0.001	2	8.68	0.002	2	0.06	0.002	2	9.74	0.000
*Persoonia levis*	3	206.27	0.068	6	3.60	0.130	2	45.59	0.002	6	5.68	0.068	6	5.87	0.004	2	0.30	0.000	3	1.08	0.001	2	8.16	0.002	2	0.05	0.000	2	7.66	0.000
*Dodonaea viscosa*	28	6.89	0.189	26	2.63	0.320	6	9.29	0.054	18	6.61	0.107	19	19.23	0.058	16	1.20	0.099	9	2.61	0.071	6	11.64	0.051	1	0.09	0.000	6	4.53	0.046
*Pimelea linifolia*	5	2.85	0.114	6	1.19	0.134	2	12.64	0.002	4	13.76	0.121	6	14.39	0.022	5	0.50	0.034	3	0.85	0.003	3	7.91	0.002	2	0.11	0.002	3	8.57	0.030
*Quercus ilex*	7	2241.03	0.068	14	17.41	0.285	1	22.75		283	6.24	0.109	449	14.00	0.070	297	0.88	0.129	30	1.89	0.129	20	7.24	0.181	18	0.05	0.110	11	2.68	0.209
*Quercus robur*	8	3219.44	0.155	33	26.48	0.233	2	6.01	0.001	103	14.07	0.090	227	23.35	0.097	190	1.78	0.151	48	1.67	0.153	3	7.40	0.001	2	0.08	0.010	3	5.57	0.035
*Fagus sylvatica*	16	194.92	0.120	23	30.96	0.189	2	6.01	0.001	273	15.39	0.161	260	22.61	0.078	148	1.42	0.108	205	1.21	0.149	6	5.18	0.160	10	0.08	0.190	3	6.77	0.010
*Simarouba amara*	5	221.99	0.243	3	34.28	0.020	2	11.63	0.040	6	8.40	0.183	5	20.08	0.109	4	0.73	0.094	3	2.30	0.132	1	13.84	0.000	1	0.08	0.000	1	4.52	0.000
*Synoum glandulosum*	6	197.77	0.126	10	3.80	0.307	2	11.75	0.001	10	11.68	0.065	6	16.22	0.014	5	0.87	0.022	3	1.46	0.002	2	6.46	0.000	2	0.07	0.002	3	4.54	0.011
*Eucalyptus socialis*	4	0.81	0.031	7	6.94	0.186	2	28.78	0.001	6	3.49	0.012	15	10.83	0.059	14	0.54	0.096	9	3.67	0.024	2	16.23	0.000	2	0.05	0.000	2	4.45	0.001
*Brachychiton populneus*	6	108.17	0.217	8	7.76	0.221	3	13.21	0.001	8	8.70	0.070	11	16.99	0.045	10	0.91	0.040	6	2.13	0.046	4	8.49	0.070	4	0.06	0.103	4	3.85	0.044
*Larix decidua*	9	6.42	0.099	20	37.65	0.184	5	6.01	0.001	90	9.73	0.063	89	19.81	0.072	76	1.79	0.156	12	2.10	0.112	5	5.42	0.161	5	0.06	0.212	5	3.13	0.194
*Picea abies*	23	6.37	0.078	24	40.02	0.246	3	88.85	0.109	146	4.45	0.134	954	12.40	0.081	812	1.42	0.134	109	3.07	0.116	5	7.67	0.071	5	0.03	0.017	5	2.07	0.117
*Pinus sylvestris*	29	7.32	0.133	31	25.38	0.244	5	27.71	0.016	430	4.92	0.103	1422	13.06	0.088	1245	1.30	0.117	359	2.80	0.121	6	10.97	0.031	6	0.04	0.021	6	2.73	0.046
*Pseudotsuga menziesii*	25	11.36	0.054	29	61.79	0.184	2	64.68	0.001	10	6.30	0.153	105	12.29	0.079	82	1.69	0.138	5	1.58	0.135	35	9.12	0.158	4	0.03	0.104	4	2.99	0.091

SD is based on log_10_-transformed trait data, after exclusion of duplicates and outliers, including data derived under experimental growth conditions. Numbers in brackets along with names of plant functional types characterize the numbers of species attributed to the respective PFT. Plant species were selected to provide examples from different functional types and with entries for each of the 10 traits.

SD, standard deviation within group; SD between, standard deviation between groups; *n*, number of entries; nsp, *n*/sp and *n*/PFT, number of species vs. number of mean number of entries per species and PFT, mean values, calculated as arithmetic mean on log-scale and retransformed to original scale, Sign. *P*, significance level for difference between means for PFTs and species; Traits, seed mass (mg); plant height, maximum plant height (m); LL, leaf lifespan (month); SLA, specific leaf area (mm^2^ mg^−1^); *N*_m_, leaf nitrogen content per dry mass (mg g^−1^); *P*_m_, leaf phosphorus content per dry mass (mg g^−1^); *N*_a_, leaf nitrogen content per area (g m^−2^), 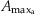, light saturated photosynthetic rate per leaf area (μmol m^−2^ s^−1^); 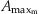, light saturated photosynthetic rate per dry mass (μmol g^−1^ s^−1^), 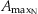, light saturated photosynthetic rate per leaf nitrogen content (μmol g^−1^ s^−1^).

The evaluation of the two tenets of comparative ecology and vegetation modelling focuses on 10 traits that are central to leading dimensions of trait variation or that are physiologically relevant and closely related to parameters used in vegetation modelling (Westoby *et al.*, 2002; Wright *et al.*, 2004): plant height, seed mass, specific leaf area (one-sided leaf area per leaf dry mass, *SLA*), leaf longevity, leaf nitrogen content per leaf dry mass (*N*_m_) and per leaf area (*N*_a_), leaf phosphorus content per leaf dry mass (*P*_m_) and maximum photosynthetic rate per leaf area (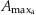), per leaf dry mass (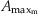) and per leaf nitrogen content (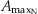). As for the relevance of the 10 selected traits: plant height was considered relevant for vegetation carbon storage capacity; seed mass was considered relevant for plant regeneration strategy; leaf longevity was considered relevant for trade-off between leaf carbon investment and gain; SLA for links of light capture (area based) and plant growth (mass based); leaf N and P content: link of carbon and respective nutrient cycle; photosynthetic rates expressed per leaf area, dry mass and N content for links of carbon gain to light capture, growth and nutrient cycle. Although we realize the relevance of traits related to plant–water relations, we did not feel comfortable to include traits such as maximum stomatal conductance or leaf water potential into the analyses for the lack of sufficient coverage for a substantial number of species. For each of the 10 traits, we quantified variation across species and PFTs in three ways: (1) Differences between mean values of species and PFTs were tested, based on one-way anova. (2) Variation within species, in terms of standard deviation (SD), was compared with variation between species (same for PFTs). (3) The fraction of variance explained by species and PFT *R*^2^ was calculated as one minus the residual sum of squares divided by the total sum of squares.

We observed large variation in SD within species if the number of observations per species was small (see funnel plot in Appendix S1). With an increasing number of observations, SD within species approached an average, trait specific level. To avoid confounding effects due to cases with very few observations per species, only species with at least five trait entries were used in statistical analyses (with exception of leaf longevity, where two entries per species were taken as the minimum number because species with multiple entries were very rare). The number of measurements per PFT was sufficient in all cases. Statistical analyses were performed in r (R Development Core Team, 2009).

## Results

### Data coverage in the TRY database

As of March 31, 2011 the TRY data repository contains 2.88 million trait entries for 69 000 plant species, accompanied by 3.0 million ancillary data entries [not all data from the databases listed in Table 1 and summarized in Table 2 could be used in the subsequent analyses, because some recently contributed datasets were still being checked and cleaned in the data staging area (see Fig. 1)]. About 2.8 million of the trait entries have been measured in natural environment, <100 000 in experimental conditions (e.g. glasshouse, climate or open-top chambers). About 2.3 million trait entries are for quantitative traits, while 0.6 million entries are for qualitative traits (Table 2). Qualitative traits, like plant growth form, are often treated as distinct and invariant within species (even though in some cases they are more variable than studies suggest, e.g. flower colour or dispersal mode), and they are often used as covariates in analyses, as when comparing evergreen vs. deciduous (Wright *et al.*, 2005) or resprouting vs. nonresprouting plants (Pausas *et al.*, 2004). The qualitative traits with the highest species coverage in the TRY dataset are the five traits used for PFT classification and leaf compoundness: woodiness (44 000 species), plant growth form (40 000), leaf compoundness (35 000), leaf type (34 000), photosynthetic pathway (32 000) and leaf phenology type (16 000); followed by N-fixation capacity (11 000) and dispersal syndrome (10 000). Resprouting capacity is noted for 3000 species (Description of qualitative traits: Plant dispersal syndrome: dispersed by wind, water, animal; N-fixation capacity: able/not able to fix atmospheric *N*_2_; leaf compoundness: simple versus compound, resprouting capacity: able/not able to resprout).

The quantitative traits with the highest species coverage are seed size (27 000 species), plant height (18 000), leaf size (17 000), wood density (12 000), SLA (9000), plant longevity (8000), leaf nitrogen content (7000) and leaf phosphorus content (5000). Leaf photosynthetic capacity is characterized for more than 2000 species. Some of these traits are represented by a substantial number of entries per species, e.g. *SLA* has on average 10 entries per species, leaf N, P and photosynthetic capacity have about eight resp. five entries per species, with a maximum of 1470 entries for leaf nitrogen per dry mass (*N*_m_) for *Pinus sylvestris*.

**Fig. 2:**
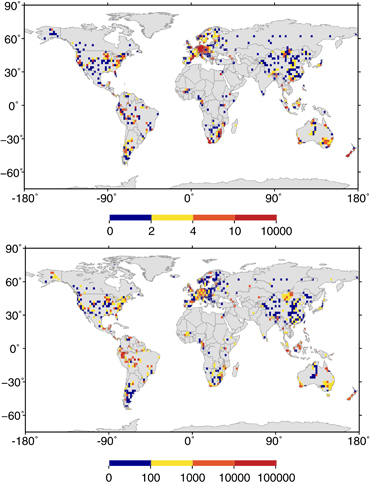
Data density of georeferenced trait entries. Top, number of sites per 2 × 2° grid cell; bottom, number of trait entries per grid cell.

**Fig. 3:**
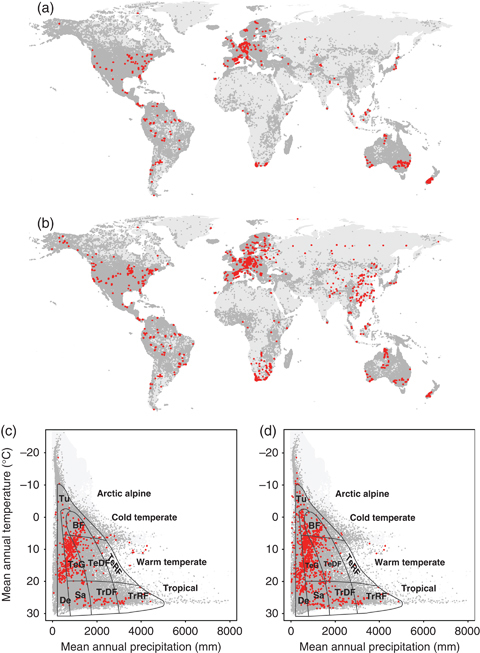
Data density for (a) specific leaf area (SLA) (1862 sites) and (b) leaf nitrogen content per dry mass (3458 sites), and data density in climate space: (c) *SLA* and (d) leaf nitrogen content per dry mass (*N*_m_). Red: geo-referenced measurement sites in the TRY database; dark grey: distribution of entries in the GBIF database (Global Biodiversity Information Facility, http://www.gbif.org) for species characterized by entries of SLA or leaf nitrogen content per dry mass in the TRY database; light grey: continental shape, respectively, all entries in the GBIF database in climate space. Mean annual temperature and mean annual precipitation are based on CRU gridded climate data (CRU: Climate Research Unit at the University of East Anglia, UK: http://www.cru.uea.ac.uk). Climate space overlaid by major biome types of the world following Whittaker *et al.* (1975): Tu, Tundra; BF, Boreal Forest; TeG, Temperate Grassland; TeDF, Temperate Deciduous Forest; TeRF, Temperate Rain Forest; TrDF, Tropical Deciduous Forest; TrRF, Tropical Rain Forest; Sa, Savanna; De, Desert. Biome boundaries are approximate.

About 40% of the trait entries (1.3 million) are georeferenced, allowing trait entries to be related to ancillary information from external databases such as climate, soil, or biome type. Although latitude and longitude are often recorded with high precision, the accuracy is unknown. The georeferenced entries are associated with 8502 individual measurement sites, with sites in 746 of the 4200 2 × 2° land grid cells of e.g. a typical climate model (Fig. 2). Europe has the highest density of measurements, and there is good coverage of some other regions, but there are obvious gaps in boreal regions, the tropics, northern and central Africa, parts of South America, southern and western Asia. In tropical South America, the sites fall in relatively few grid cells, but there are high numbers of entries per cell. This is an effect of systematic sampling efforts by long-term projects such as LBA (The Large Scale Biosphere-Atmosphere Experiment in Amazonia: http://www.lba.inpa.gov.br/lba) or RAINFOR (Amazon Forest Inventory Network: http://www.geog.leeds.ac.uk/projects/rainfor). For two individual traits, the spatial coverage is shown in Fig. 3. Here we additionally provide coverage in climate space, identifying biomes for which we lack data (e.g. temperate rainforests). More information about data coverage of individual traits is available on the website of the TRY initiative (http://www.try-db.org).

### General pattern of trait variation: test for normality

For 52 traits, the coverage of database entries was sufficient to quantify general pattern of density distributions in terms of skewness and kurtosis, and to apply the Jarque–Bera test for normality (Table 3). On the original scale all traits but one are positively skewed, indicating distributions tailed to high values. After log-transformation, the distributions of 20 traits are still positively skewed, while 32 traits show slightly negative skewness. For 49 of the 52 traits, the Jarque–Bera test indicates an improvement of normality by log-transformation of trait values – only for three traits normality was deteriorated (leaf phenolics, tannins and carbon content per dry mass; Table 3). The distribution of leaf phenolics and tannins content per dry mass is in between normal and log-normal: positively skewed on the original scale, negatively skewed on log-scale. Leaf carbon content per dry mass has a theoretical range from 0 to 1000 mg g^−1^. The mean value, about 476 mg g^−1^, is in the centre of the theoretical range, and the variation of trait values is small (Table 4).

**Table 3:** Statistical properties for the density distributions of 52 traits with substantial coverage and a test for deviation from normality, on the original scale and after log-transformation of trait values

Trait	Number of entries	Original scale				Logarithmic scale				Change of normality
	
		Skewness	Kurtosis	JB test	*P*-value	Skewness	Kurtosis	JB test	*P*-value	
**Seed dry mass**	**53 744**	**123.02**	**19 457.16**	**8.E+11**	**<2.20E−16**	**0.53**	**0.42**	**2915**	**<2.20E−16**	**8.E+11**
Leaf dry mass	26 220	161.48	26 118.88	7.E+11	<2.20E−16	−0.45	0.90	1748	<2.20E−16	7.E+11
Leaf area	76 883	65.47	6990.13	2.E+11	<2.20E−16	−0.54	0.02	3798	<2.20E−16	2.E+11
Conduit (vessel and tracheid) density	5454	68.93	4968.04	6.E+09	<2.20E−16	−0.03	−0.43	43	<2.20E−16	6.E+09
Leaf Fe content per dry mass	3128	31.84	1084.72	2.E+08	<2.20E−16	1.51	8.78	11 229	<2.20E−16	2.E+08
Releasing height	19 668	13.86	292.85	7.E+07	<2.20E−16	0.70	2.33	6068	<2.20E−16	7.E+07
Leaf Mn content per dry mass	3273	12.04	222.70	6 842 757	<2.20E−16	−0.02	−0.51	35	2.41E−08	6 842 722
Seed length	9336	7.41	89.35	3 191 250	<2.20E−16	0.31	0.47	239	<2.20E−16	3 191 011
Whole leaf nitrogen content	1006	12.84	248.60	2 618 135	<2.20E−16	−0.53	0.08	48	4.06E−11	2 618 087
Leaf Na content per dry mass	3180	9.55	126.32	2 162 452	<2.20E−16	0.19	0.79	100	<2.20E−16	2 162 352
**Specific leaf area (SLA)**	**4 8142**	**2.85**	**27.49**	**1 581 085**	**<2.20E−16**	**-0.54**	**1.06**	**4555**	**<2.20E−16**	**1 576 530**
**Leaf phosphorus content per dry mass (P_m_)**	**17 920**	**3.58**	**42.89**	**1 412 132**	**<2.20E−16**	**−0.38**	**0.98**	**1155**	**<2.20E−16**	**1 410 977**
Leaf phosphorus content per area	5290	5.33	71.12	1 139 938	<2.20E−16	−0.04	0.75	125	<2.20E−16	1 139 813
Leaf Zn content per dry mass	3278	8.04	84.86	1 018 873	<2.20E−16	1.35	2.55	1880	<2.20E−16	1 016 993
Maximum plant longevity	2006	7.31	97.69	815 546	<2.20E−16	−0.91	1.40	442	<2.20E−16	815 104
**Leaf lifespan (longevity)**	**1654**	**7.26**	**91.59**	**592 617**	**<2.20E−16**	**0.31**	**−0.35**	**34**	**4.30E−08**	**592 583**
Whole leaf phosphorus content	444	10.23	141.53	378 307	<2.20E−16	−0.27	−0.34	7	0.02529	378 299
Leaf K content per dry mass	4144	4.09	33.47	204 954	<2.20E−−16	0.09	0.33	24	6.64E−06	204 930
Leaf Al content per dry mass	3448	5.14	35.08	191 974	<2.20E−16	1.13	1.01	876	<2.20E−16	191 098
Leaf nitrogen/phosphorus (N/P) ratio	11 612	3.03	17.65	168 595	<2.20E−16	0.25	0.41	199	<2.20E−16	168 396
Seed terminal velocity	1178	3.91	50.26	126 989	<2.20E−16	−0.45	−0.77	69	9.99E−16	126 920
Leaf mechanical resistance: tear resistance	758	6.53	59.82	118 402	<2.20E−16	0.86	1.11	132	<2.20E−16	118 270
Leaf thickness	2934	4.24	29.88	117 951	<2.20E−16	0.77	0.71	351	<2.20E−16	117 600
**Maximum Plant height**	**28 248**	**2.35**	**6.99**	**83 464**	**<2.20E−16**	**0.11**	**−0.89**	**983**	**<2.20E−16**	**82 481**
Leaf respiration per dry mass	2234	4.28	24.65	63 393	<2.20E−16	0.29	0.62	66	4.77E−15	63 327
Wood phosphorus content per dry mass	1056	4.93	35.87	60 888	<2.20E−16	0.71	0.31	94	<2.20E−16	60 794
**Leaf nitrogen content per area (N_a_)**	**13 528**	**1.73**	**8.25**	**45 047**	**<2.20E−16**	**−0.27**	**0.34**	**224**	**<2.20E−16**	**44 823**
Leaf Mg content per dry mass	3485	2.55	15.68	39 460	<2.20E−16	−0.14	0.13	14	0.001098	39 446
Conduit (vessel and tracheid) area	3050	3.31	15.89	37 636	<2.20E16	−0.24	−0.09	31	2.15E−07	37 605
Leaf S content per dry mass	1092	4.60	24.78	31 788	<2.20E−16	1.45	4.21	1189	<2.20E−16	30 600
Leaf Ca content per dry mass	3755	2.11	10.09	18 721	<2.20E−16	−0.83	1.19	656	<2.20E−16	18 065
**Leaf nitrogen content per dry mass (N_m_)**	**35 862**	**1.21**	**2.33**	**16 905**	**<2.20E−16**	**−0.22**	**−0.38**	**407**	**<2.20E−16**	**16 498**
Vessel diameter	3209	2.61	9.61	15 977	<2.20E−16	0.27	−0.35	54	1.83E−12	15 923
Conduit lumen area per sapwood area	2280	2.41	9.75	11 243	<2.20E−16	−0.37	0.97	140	<2.20E−16	11 102
Canopy height observed	40 510	1.25	1.04	12 416	<2.20E−16	−0.15	−1.22	2654	<2.20E−16	9762
Leaf dry matter content (LDMC)	17 339	1.10	2.68	8693	<2.20E−16	−0.46	0.85	1141	<2.20E−16	7551
Leaf respiration per dry mass at 25°C	1448	2.70	9.24	6907	<2.20E−16	0.49	0.63	82	<2.20E−16	6825
Stomatal conductance per leaf area	1093	2.39	10.69	6250	<2.20E−16	−0.73	1.27	171	<2.20E−16	6079
**Photosynthesis per leaf dry mass (** 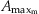 **)**	**2549**	**2.09**	**6.01**	**5699**	**<2.20E−16**	**−0.36**	**0.13**	**58**	**2.85E−13**	**5642**
Leaf Si content per dry mass	1057	2.35	9.82	5219	<2.20E−16	−0.54	0.84	82	<2.20E−16	5137
Vessel element length	3048	1.63	5.12	4668	<2.20E−16	−0.28	0.35	55	9.89E−13	4613
Wood nitrogen content per dry mass	1259	2.22	8.24	4591	<2.20E−16	0.33	0.15	24	5.93E−06	4567
**Photosynthesis per leaf area (** 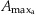 **)**	**3062**	**1.49**	**3.20**	**2436**	**<2.20E−16**	**−0.63**	**1.32**	**422**	**<2.20E−16**	**2014**
Leaf K content per area	240	3.12	12.28	1898	<2.20E−16	0.37	0.55	9	0.01393	1890
Leaf carbon/nitrogen (C/N) ratio	2615	0.95	1.99	824	<2.20E−16	−0.12	−0.18	10	0.008102	815
Wood density	26 414	0.44	−0.15	887	<2.20E−16	−0.17	−0.40	298	<2.20E−16	589
Leaf density	1463	1.01	2.59	655	<2.20E−16	−0.56	0.79	115	<2.20E−16	540
Root nitrogen content per dry mass	1263	1.33	1.35	466	<2.20E−16	−0.05	−0.54	16	0.0003217	450
Leaf respiration per area	1303	1.22	2.00	542	<2.20E−16	−0.79	1.80	312	<2.20E−16	230
Leaf phenolics content per dry mass	471	0.52	0.21	22	1.90E−05	−1.16	1.41	144	<2.20E−16	−123
Leaf carbon content per dry mass	8140	−0.07	0.03	7	2.67E−02	−0.32	0.08	144	<2.20E−16	−137
Leaf tannins content per dry mass	409	1.40	2.87	274	<2.20E−16	−2.10	6.89	1109	<2.20E−16	−835
**Average**		**12.25**	**1165.87**			**−0.05**	**0.83**			
**RMSE**		**2.44**	**13.37**			**0.29**	**0.40**			

Results based on dataset after excluding obvious errors, but before detection of outliers. Skewness, measure of the asymmetry of the density distribution (0 in case of normal distribution; <0, left-tailed distribution; >0, right-tailed distribution); Kurtosis, measure of the ‘peakedness’ of the density distribution (here presented as excess kurtosis: 0, in case of normal distribution; <0, wider peak around the mean; >0, a more acute peak around the mean); JB test, result of Jarque–Bera test for departure from normality (0 for normal distribution; >0 for deviation from normal distribution); *P*-value, probability of obtaining a test statistic at least as extreme as the observed, assuming the null hypothesis, here the data are normal distributed, is true (on the original scale, resp. after log-transformation, >0.5 in case of normality accepted at 95% confidence); change of normality, difference between results of Jarque–Bera test on the original scale and after log-transformation of trait data (>0, improvement of normality by log-transformation; <0, deterioration of normality by log-transformation); RMSE, root mean squared error; bold: traits for which we quantified the fraction of variance explained by species and PFT.

**Table 4:** Mean values and ranges for 52 traits with substantial coverage, based on individual trait entries, after exclusion of outliers and duplicates

Trait	Number of entries	Unit	Mean value	SD_lg_	2.5% Quantile	Median	97.5% Quantile
**Seed dry mass**	**49 837**	**mg**	**2.38**	**1.08**	**0.02**	**1.95**	**526**
Canopy height observed	37 516	m	1.62	0.92	0.04	1.5	30
Whole leaf phosphorus content	426	mg	0.0685	0.83	0.0018	0.08	1.96
Leaf area	71 929	mm^2^	1404.0	0.81	25	2025	36 400
**Maximum plant height**	**26 625**	**m**	**1.84**	**0.78**	**0.1**	**1.25**	**40**
Leaf dry mass	24 663	mg	38.9	0.78	0.96	43.5	1063.9
Whole leaf nitrogen content	961	mg	1.31	0.77	0.03	1.69	27.6
Conduit (vessel and tracheid) area	2974	mm^2^	0.00349	0.63	0.00024	0.0032	0.04
Leaf Mn content per dry mass	3159	mg g^−1^	0.189	0.58	0.01	0.19	2.13
Maximum plant longevity	1854	year	155.8	0.55	6.22	175	1200
Leaf Al content per dry mass	3203	mg g^−1^	0.128	0.55	0.02	0.1	4.49
Leaf Na content per dry mass	3086	mg g^−1^	0.200	0.55	0.01	0.2	3.24
Conduit (vessel and tracheid) density	5301	mm^−2^	37.6	0.54	4	38	380
Seed terminal velocity	1108	m s^−1^	1.08	0.42	0.17	1.4	4.69
Releasing height	18 472	m	0.347	0.42	0.05	0.35	2
**Leaf lifespan (longevity)**	**1540**	**month**	**9.40**	**0.41**	**2**	**8.5**	**60**
Leaf tannins content per dry mass*	394	%	2.01	0.41	0.19	2.35	8.04
Wood phosphorus content per dry mass	1016	mg g^−1^	0.0769	0.37	0.02	0.05	0.56
Leaf respiration per dry mass	2005	μmol g^−1^ s^−1^	0.0097	0.36	0.0025	0.0097	0.04
Seed length	8770	mm	1.80	0.34	0.4	1.8	9
**Photosynthesis per leaf dry mass (** 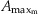 **)**	**2384**	**μmol g^−1^ s^−1^**	**0.115**	**0.34**	**0.02**	**0.12**	**0.49**
Leaf mechanical resistance: tear resistance	722	N mm^−1^	0.814	0.34	0.19	0.76	5.11
Leaf Ca content per dry mass	3594	mg g^−1^	9.05	0.34	1.57	9.83	34.7
Vessel diameter	3102	μm	51.4	0.32	15	50	220
Stomatal conductance per leaf area	1032	mmol m^−1^ s^−1^	241.0	0.31	52.4	243.7	895.7
Root nitrogen content per dry mass	1158	mg g^−1^	9.67	0.31	2.6	9.3	36.1
Leaf Si content per dry mass	1027	mg g^−1^	0.163	0.29	0.04	0.17	0.53
Leaf Zn content per dry mass	3080	mg g^−1^	0.0226	0.28	0.0065	0.02	0.1
Leaf respiration per dry mass at 25°C	1305	μmol g^−1^ s^−1^	0.0092	0.28	0.0035	0.0082	0.03
Leaf K content per dry mass	3993	mg g^−1^	8.44	0.27	2.56	8.3	28.2
**Photosynthesis per leaf N content (** 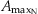 **)**	**3074**	**μmol g^−1^ s^−1^**	**10.8**	**0.27**	**1.59**	**6.32**	**19.2**
Leaf phenolics content per dry mass*	454	%	12.1	0.26	2.43	11.9	25.1
**Specific leaf area (SLA)**	**45 733**	**mm^2^ mg^−1^**	**16.6**	**0.26**	**4.5**	**17.4**	**47.7**
Leaf K content per area	231	g m^−2^	0.760	0.26	0.24	0.72	2.60
Leaf Mg content per dry mass	3360	mg g^−1^	2.61	0.25	0.83	2.64	8.0
Leaf Fe content per dry mass	3040	mg g^−1^	0.077	0.25	0.02	0.07	0.26
**Photosynthesis per leaf area (** 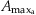 **)**	**2883**	**μmol m^−2^ s^−1^**	**10.3**	**0.24**	**3.28**	**10.5**	**29**
Leaf respiration per area	1201	μmol m^−2^ s^−1^	1.19	0.24	0.38	1.2	3.4
**Leaf phosphorus content per dry mass (P_m_)**	**17 057**	**mg g^−1^**	**1.23**	**0.24**	**0.40**	**1.25**	**3.51**
Leaf thickness	2815	mm	0.211	0.24	0.08	0.19	0.7
Conduit lumen area per sapwood area	2210	mm^2^ mm^−2^	0.137	0.23	0.04	0.14	0.37
Leaf phosphorus content per area	5083	g m^−2^	0.104	0.23	0.03	0.1	0.28
Vessel element length	2964	μm	549.5	0.21	200	555	1350
Leaf nitrogen/phosphorus (N/P) ratio	11 200	g g^−1^	12.8	0.21	5.33	12.6	33.2
**Leaf nitrogen content per area (N_a_)**	**12 860**	**g m^−2^**	**1.59**	**0.19**	**0.64**	**1.63**	**3.6**
Wood nitrogen content per dry mass	1210	mg g^−1^	1.20	0.19	0.55	1.21	2.95
Leaf S content per dry mass	1023	mg g^−1^	1.66	0.18	0.78	1.59	4.75
**Leaf nitrogen content per dry mass (N_m_)**	**33 880**	**mg g^−1^**	**17.4**	**0.18**	**7.99**	**17.4**	**38.5**
Leaf dry matter content (LDMC)	16 185	g g^−1^	0.213	0.17	0.1	0.21	0.42
Leaf density	1372	g cm^−3^	0.426	0.15	0.2	0.43	0.77
Leaf carbon/nitrogen (C/N) ratio	2498	g g^−1^	23.4	0.14	12.39	23.5	42.2
Wood density	26 391	mg mm^−3^	0.597	0.12	0.33	0.6	0.95
Leaf carbon content per dry mass*	7856	mg g^−1^	476.1	0.03	404.5	476.3	540.8

* Mean values for leaf phenolics, tannins and carbon content were calculated on the original scale, the SD is, provided on log-scale, for comparability.

Values for 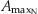 were calculated based on database entries for *A*_max_ and leaf N content per area, resp. dry mass. Mean values have been calculated as arithmetic means on a logarithmic scale and retransformed to original scale. SD, standard deviation on log_10_-scale. Traits are sorted by decreasing SD. Bold: traits for which we quantified the fraction of variance explained by species and PFT (cf. Table 5, Fig. 5).

**Fig. 4:**
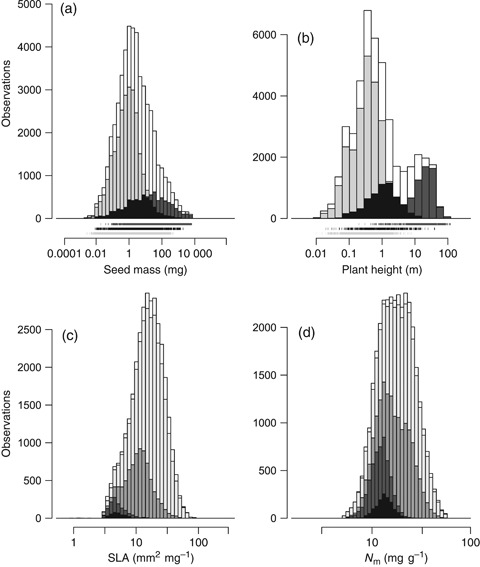
Examples of trait frequency distributions for four ecologically relevant traits (Westoby, 1998; Wright *et al.*, 2004). Upper panels: (a) seed mass and (b) plant height for all data and three major plant growth forms (white, all database entries; light grey, herbs/grasses; dark grey, trees; black, shrubs). Rug-plots provide data ranges hidden by overlapping histograms. Lower panels: (c) Specific leaf area (*SLA*) and (d) leaf nitrogen content per dry mass [*N*_m_, white, all database entries excluding outliers (including experimental conditions); light grey, database entries from natural environment (excluding experimental conditions); medium grey, growth form trees; dark grey, PFT needle-leaved evergreen; black, *Pinus sylvestris*].

Nevertheless, according to the Jarque–Bera test, also on a logarithmic scale all traits show some degree of deviation from normal distributions (indicated by small *P*-values, Table 3). Seed mass, for example, is still positively skewed after log-transformation (Table 3). This is due to substantial differences in the number of database entries and seed masses between grasses/herbs, shrubs and trees (Fig. 4a). Maximum plant height in the TRY database has a strong negative kurtosis after log-transformation (Table 3). This is due to a bimodal distribution: one peak for herbs/grass and one for trees (Fig. 4b). The number of height entries for shrubs is comparatively small – which may be due to a small number or abundance of shrub species *in situ* (i.e. a real pattern) but is more likely due to a relative ‘undersampling’ of shrubs (i.e. an artefact of data collection). Within the growth forms herbs/grass and shrubs, height distribution is approximately log-normal. For trees the distribution is skewed to low values, because there are mechanical constrictions to grow taller than 100 m. The distribution of *SLA* after log-transformation is negatively skewed with positive kurtosis (Table 3) – an imprint of needle-leaved trees and shrubs besides the majority of broadleaved plants (Fig. 4c). The distribution of leaf nitrogen content per dry mass after log-transformation has small skewness, but negative kurtosis (Table 3) – the data are less concentrated around the mean than normal (Fig. 4d). In several cases, sample size is sufficient to characterize the distribution at different levels of aggregation, down to the species level. Again we find approximately log-normal distributions (e.g. *SLA* and *N*_m_ for *Pinus sylvestris*; Fig. 4c and d).

### Ranges of trait variation

There are large differences in variation across traits (Table 4). The standard deviation (SD) expressed on a logarithmic scale ranges from 0.03 for leaf carbon content per dry mass (resp. about 8% on the original scale) to 1.08 for seed mass (resp. −95% and +1100% on the original scale). Note two characteristics of SD on the logarithmic scale: (1) it corresponds to an asymmetric distribution on the original scale: small range to low values, large range to high values; (2) it can be compared directly across traits. For more information, see supporting information Appendix S2. Leaf carbon content per dry mass, stem density and leaf density show the lowest variation, followed by the concentration of macronutrients (nitrogen, phosphorus), fluxes and conductance (photosynthesis, stomatal conductance, respiration), the concentration of micronutrients (e.g. aluminium, manganese, sodium), traits related to length (plant height, plant and leaf longevity), and traits related to leaf area. Mass-related traits show the highest variation (seed mass, leaf dry mass, N and P content of the whole leaf – in contrast to concentration per leaf dry mass or per leaf area). The observations reveal a general tendency towards higher variation with increasing trait dimensionality (length <area <mass; for more information, see Appendix S3).

### Tenet 1: Aggregation at the species level represents the major fraction of trait variation

There is substantial intraspecific variation for each of the 10 selected traits (Table 5): for single species the standard deviation is above 0.3 on logarithmic scale, e.g. SD=0.34 for maximum plant height of *Phyllota phyllicoides* (−55% and +121% on the original scale), but based on only six observations and SD=0.32 in case of *Dodonaea viscosa* (*n*=26). The SD of *N*_m_ for *Poa pratensis* is 0.17 (*n*=63), which is almost equal to the range of all data reported for this trait, but this is an exceptional case. The trait and species with the most observations is nitrogen content per dry mass for *Pinus sylvestris* with 1470 entries (SD=0.088, −18% and +22%). The variation in this species spans almost half the overall variation observed for this trait (SD=0.18), covering the overall mean (Fig. 4d). For several trait-species combinations, the number of measurements is high enough for detailed analyses of the variation within species (e.g. on an environmental gradient).

**Fig. 5:**
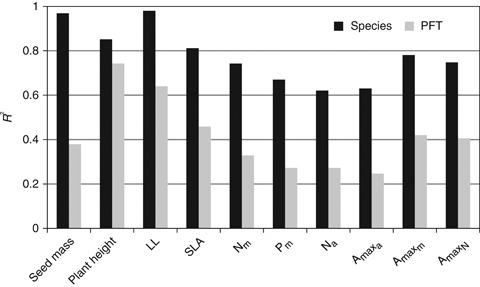
Fraction of variance explained by plant functional type (PFT) or species for 10 relevant and well-covered traits. *R*^2^, fraction of explained variance; Traits: *Seed mass*, seed dry mass; *Plant height*, maximum plant height; *LL*, leaf longevity; *SLA*, specific leaf area; *N*_m_, leaf nitrogen content per dry mass; *P*_m_, leaf phosphorus content per dry mass; *N*_a_, leaf nitrogen content per area; 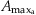, maximum photosynthesis rate per leaf area; 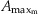, maximum photosynthesis rate per leaf dry mass; 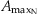, maximum photosynthesis rate per leaf nitrogen content.

The mean SD at the species-level is highest for plant height (0.18) and lowest for leaf longevity (0.03, but few observations per species, Table 5). For all ten traits the mean SD within species is smaller than the SD between species mean values (Table 5). Based on anova, mean trait values are significantly different between species: at the global scale 60–98% of trait variance occurs interspecific (between species, Fig. 5). Nevertheless, for three traits (*P*_m_, *N*_a_, 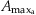) almost 40% of the variance occurs intraspecific (within species, Fig. 5).

### Tenet 2: Basic PFTs capture a sufficiently important fraction of trait variation to represent functional diversity

For all 10 traits, the PFT mean values are significantly different between PFTs (Table 5). Four traits show larger variation between PFT mean values than within PFTs (plant height, seed mass, leaf longevity, 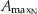), two traits show similar variation between PFT means and within PFTs (*SLA*, 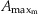). As a consequence, more than 60% of the observed variance occurs between PFTs for plant height and leaf longevity, and about 40% of the variation occurs between PFTs for seed mass, *SLA*, 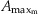 and 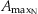 (Fig. 5). The high fraction of explained variance for these six traits reflects the definition of PFTs based on the closely related qualitative traits: plant growth form, leaf phenology (evergreen/deciduous), leaf type (needle-leaved/broadleaved) and photosynthetic pathway (C3/C4). For theses traits, PFTs such as those commonly used in vegetation models, capture a considerable fraction of observed variation with relevant internal consistency. However, for certain traits the majority of variation occurs within PFTs: four traits show smaller variation between than within PFTs, causing substantial overlap across PFTs (*N*_m_, *N*_a_, *P*_m_, 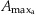). In these cases only about 20–30% of the variance is explained by PFT, and about 70–80% of variation occurs within PFTs.

### Representation of trait variation in the context of global vegetation models

To demonstrate how the observed trait variation is represented in global vegetation models, we first compare observed trait ranges of *SLA* to parameter values for *SLA* used in 12 global vegetation models; then we compare observed trait ranges of *N*_m_ with state variables of nitrogen concentration calculated within the dynamic global vegetation model O-CN (Zaehle & Friend, 2010).

**Fig. 6:**
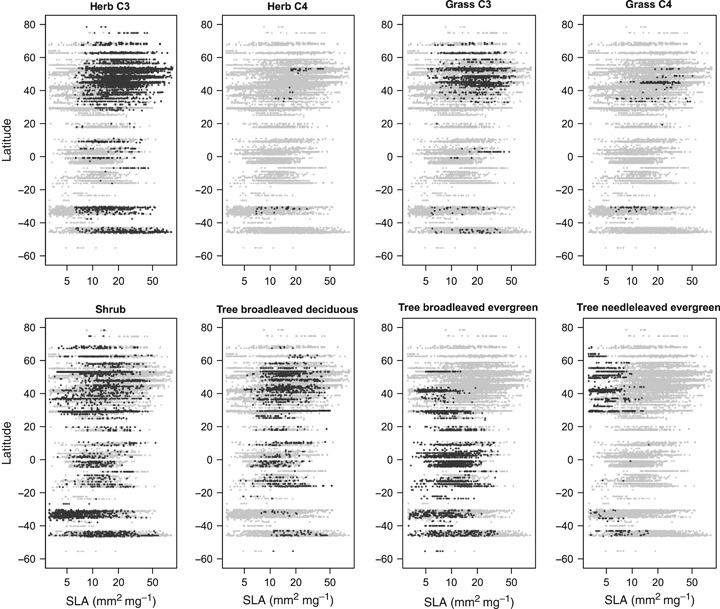
Worldwide range in specific leaf area (*SLA*) along a latitudinal gradient for the main plant functional types. Grey, all data; black, data for the plant functional group (PFT) under scrutiny.

**Fig. 7:**
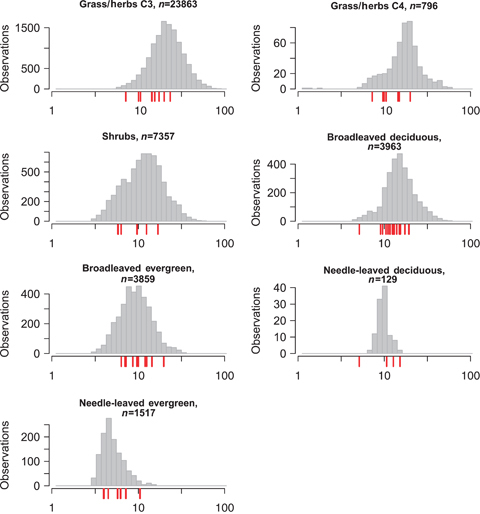
Frequency distributions of specific leaf area (*SLA*, mm^2^ mg^−1^) values (grey histograms) compiled in the TRY database and parameter values for SLA (red dashes) published in the context of the following global vegetation models: Frankfurt Biosphere Model (Ludeke *et al.*, 1994; Kohlmaier *et al.*, 1997), SCM (Friend & Cox, 1995), HRBM (Kaduk & Heimann, 1996), IBIS (Foley *et al.*, 1996; Kucharik *et al.*, 2000), Hybrid (Friend *et al.*, 1997), BIOME-BGC (White *et al.*, 2000), ED (Moorcroft *et al.*, 2001), LPJ-GUESS (Smith *et al.*, 2001), LPJ-DGVM (Sitch *et al.*, 2003), LSM (Bonan *et al.*, 2003), SEIB–DGVM (Sato *et al.*, 2007). *n*, number of SLA data in the TRY database per PFT.

Some vegetation models separate PFTs along climatic gradients into biomes, for which they assign different parameter values. A rough analysis of *SLA* along the latitudinal gradient (as a proxy for climate) indicates no major impact on *SLA* within PFT (Fig. 6), and we further jointly analyse *SLA* data by PFT. However, the range of observed trait values for *SLA* per PFT is remarkably large, except for the PFT ‘needle-leaved deciduous trees’ (Figs 6 and 7). The parameter values from most of the 12 models match moderately high density of *SLA* observations, but most are clearly different from the mean, and some parameter values are at the low ends of probabilities, surprisingly far off the mean value of observations.

**Fig. 8:**
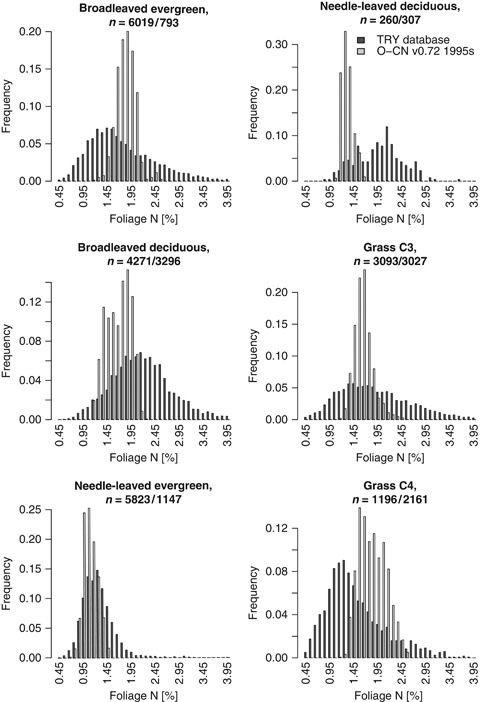
Frequency distributions of leaf nitrogen content per dry mass for major plant functional types as compiled in the TRY database compared with frequency distributions of the respective state variable calculated within the O-CN vegetation model (Zaehle & Friend, 2010). *n*, number of entries in the TRY database (left) and number of grid elements in O-CN with given PFT (right).

The range of observed trait values for *N*_m_ per PFT is also high (Fig. 8), except for the PFT ‘needle-leaved evergreen trees’. Modelled state variables are in most cases within the range of frequently observed trait values – model values for the PFT ‘needle-leaved evergreen trees’ match the observed distribution almost perfectly. Nevertheless, there are considerable differences between modelled and observed distributions: the modelled state variables are approximately normally distributed on the original scale, while the observed trait values are log-normally distributed; the range of modelled values is substantially smaller than the range of observations; and the highest densities are shifted. Apart from possible deficiencies of the O-CN model, the deviation between observed and modelled distributions may be due to inconsistencies between compiled traits and modelled state variables: trait entries in the database are not abundance-weighted with respect to natural occurrence, and they represent the variation of single measurements, while the model produces ‘community’ measures. The distribution of observed data presented here is therefore likely wider than the abundance-weighted leaf nitrogen content of communities in a given model grid cell.

## Discussion

### The TRY initiative and the current status of data coverage

The TRY initiative has been developed as a Data Warehouse to integrate different trait databases. Nevertheless, TRY does not aim to replace existing databases, but rather provides a complementary way to access these data consistently with other trait data – it facilitates synergistic use of different trait databases. Compared with a Meta Database approach, which would link a network of separate databases, the integrated database (Data Warehouse) provides the opportunity to standardize traits, add ancillary data, provide accepted species names and to identify outliers and duplicate entries. A disadvantage of the Data Warehouse approach is that some of the databases contributing to TRY are continuously being developed (see Table 2). However, these contributions to TRY are regularly updated.

The list of traits in the TRY database is not fixed, and it is anticipated that additional types of data will be added to the database in the future. Examples include sap-flow measurements, which are fluxes based on which trait values can be calculated, just as photosynthesis measurements can be used to determine parameter values of the Farquhar model (Farquhar *et al.*, 1980), and leaf venation, which has recently been defined in a consistent way and appears to be correlated with other leaf functional traits (Sack & Frole, 2006; Brodribb *et al.*, 2007; Blonder *et al.*, 2011). Ancillary data, contributed with the trait data, may include images. There is also room for expansion of the phylogenetic range of the data incorporated in the database. There is currently little information on nonvascular autotrophic cryptogams in TRY (i.e. bryophytes and lichens), despite their diversity in species, functions and ecosystem effects, and the growing number of trait measurements being made on species within these groups.

The qualitative traits with greatest coverage (more than 30 000 species for woodiness, plant growth form, leaf compoundness, leaf type, photosynthetic pathway) represent about 10% of the estimated number of vascular plant species on land. The quantitative traits with most coverage (5000–20 000 species for e.g. seed mass, plant height, wood density, leaf size, leaf nitrogen content, *SLA*) approach 5% of named plant species. Although they represent a limited set of species (5–10%), most probably they include the most abundant (dominant) species. The high number of characterized species opens up the possibility of identifying the evolutionary branch points at which large divergences in trait values occurred. Such analyses will improve our understanding of trait evolution at both temporal and spatial scales. They highlight the importance of including trait data for autotrophs representing very different branches of the Tree of Life (Cornelissen *et al.*, 2007; Lang *et al.*, 2009) in the TRY database.

For some traits, we know that many more data exist, which could potentially be added to the database. Nevertheless, for some traits the lack of data reflects difficulties in data collection. Table 2 shows some traits where species coverage is thin, most probably because the measurements are difficult or laborious. Root measurements fall into this category. Rooting depth (or more exactly, maximum water extraction depth) is among the most influential plant traits in global vegetation models, yet we have estimates for only about 0.05% of the vascular plant species. Data for other root traits is even scarcer. However, many aboveground traits correlate with belowground traits (see Kerkhoff *et al.*, 2006), so the data in TRY do give some indication about belowground traits. Apart from this, root traits are focus of current studies (Paula & Pausas, 2011). Anatomical traits also have weak coverage in general. Quantifying anatomy from microscopic cross-sections is a slow and painstaking work and there is currently no consensus on which are the most valuable variables to quantify in leaf sections, apart from standard variables such as tissue thicknesses and cell sizes, which show important correlations with physiological function, growth form and climate (Givnish, 1988; Sack & Frole, 2006; Markesteijn *et al.*, 2007; Dunbar-Co *et al.*, 2009; Hao *et al.*, 2010). An exception is wood anatomy, where TRY contains conduit densities and sizes for many species (about 7000 and 3000 species, respectively). Finally, allometric or architectural relationships that describe relative biomass allocation to leaves, stems, and roots through the ontogeny of individual plants are presently scattered across 72 different traits, each with low coverage. These traits are essential for global vegetation models and this is an area where progress in streamlining data collection is needed.

Many trait data compiled in the database were not necessarily collected according to similar or standard protocols. Indeed many fields of plant physiology and ecology lack consensus definitions and protocols for key measurements. However, progress is being made as well towards *a posteriori* data consolidation (e.g. Onoda *et al.*, 2011), as towards standardizing trait definitions and measurement protocols, e.g. via a common plant trait Thesaurus (Plant Trait Thesaurus: http://trait_ontology.cefe.cnrs.fr:8080/Thesauform/), and a handbook and website (PrometheusWiki: http://prometheuswiki.publish.csiro.au/tiki-custom_home.php) of standard definitions and protocols (Cornelissen *et al.*, 2003b; Sack *et al.*, 2010).

Information about the abiotic and biotic environment in combination with trait data is essential to allow an assessment of environmental constraints on the variation of plant traits (Fyllas *et al.*, 2009; Meng *et al.*, 2009; Ordoñez *et al.*, 2009; Albert *et al.*, 2010b; Poorter *et al.*, 2010). Some of this information has been compiled in the TRY database. However, the information about soil, climate and vegetation structure at measurement sites is not well structured, because there is no general agreement on what kind of environmental information is most useful to report in addition to trait measurements. A consensus on these issues would greatly improve the usefulness of ancillary environmental information. Geographic references should be a priority for nonexperimental data.

The number of observations or species with data for all traits declines rapidly with an increasing number of traits: fewer species have data for each trait (see Appendix S3). In cases where multivariate analyses rely on completely sampled trait-species matrices, this issue poses a significant constraint on the number of traits and/or species that can be included. Gap filling techniques, e.g. hierarchical Bayesian approaches or filtering techniques (Shan & Banerjee, 2008; Su & Khoshgoftaar, 2009) offer a potential solution. On the other hand, simulation work in phylogenetics has shown that missing data are not by themselves problematic for phylogenetic reconstruction (Wiens, 2003, 2005). Similar work could be performed in trait-based ecology, and the emerging field of ecological informatics (Recknagel, 2006) may help to identify representative trait combinations while taking incomplete information into account (e.g. Mezard, 2007).

### General pattern and ranges of trait distribution

Based on the TRY dataset, we characterized two general patterns of trait density distributions: (1) plant traits are rather log-normal than normal distributed and (2) the range of variation tends to increase with trait-dimensionality. Here the analysis did benefit from compiling large numbers of trait entries for several traits from different aspects of plant strategy. Based on the rich sampling, we could quantify simple general rules for trait distributions and still identify deviations in the individual case. The approximately log-normal distributions confirm prior reports for individual traits (e.g. Wright *et al.*, 2004) and are in agreement with general observations in biology (Kerkhoff & Enquist, 2009), although we also observe deviation from log-normal distribution, e.g. as an imprint of plant growth form or leaf type. Being approximately log-normal distributed is most probably due to the fact that plant traits often have a lower bound of zero but no upper bound relevant for the data distribution. This log-normal distribution has several implications: (1) On the original scale, relationships are to be expected multiplicative rather than additive (Kerkhoff & Enquist, 2009, see as well Appendix S2). (2) Log- or log–log scaled plots are not sophisticated techniques to hide huge variation, but the appropriate presentation of the observed distributions (e.g. Wright *et al.*, 2004). On the original scale, bivariate plots of trait distributions are to be expected heteroscedastic (e.g. Kattge *et al.*, 2009). (3) Trait related parameters and state variables in vegetation models can be assumed log-normal distributed as well, e.g. Figs 7 and 8 (Knorr & Kattge, 2005). For more details, see Appendix S2.

For several traits, we quantified ranges of variation: overall variation, intra- and interspecific variation, and variation with respect to different functional groups. Most of the trait data compiled within the TRY database have been measured within natural environments and only a small fraction comes from experiments. Therefore, the impact of experimental growth conditions on observed trait variation is probably small in most cases and the observed trait variation in the TRY database comprises primarily natural variation at the level of single organs, including variation due to different measurement methods and, of course, measurement errors. However, systematic sampling of trait variation at single locations is a relatively new approach (Albert *et al.*, 2010a, b; Baraloto *et al.*, 2010; Hulshof & Swenson, 2010; Jung *et al.*, 2010b; Messier *et al.*, 2010), and it may therefore be shown that trait variability under natural conditions is underestimated in the current dataset.

### Tenets revisited

The results presented here are a first step to illuminate two basic tenets of plant comparative ecology and vegetation modelling at a global scale: (1) The aggregation of trait data at the species level represents the major fraction of variation in trait values. At the same time, we have shown surprisingly high intraspecific variation – for some traits responsible for up to 40% of the overall variation (Table 5, Figs 4 and 5). This variation reflects genetic variation (among genotypes within a population/species) and phenotypic plasticity. Through the TRY initiative, a relevant amount of data is available to quantify and understand trait variation beyond aggregation on species level. The analysis presented here is only a first step to disentangle within- and between-species variability. It is expected that in combination with more detailed analyses the TRY database will support a paradigm shift from species to trait-based ecology.

(2) Basic PFTs, such as those commonly used in vegetation models capture a considerable fraction of observed variation with relevant internal consistency. However, for certain traits the majority of variation occurs within PFTs –responsible for up to 75% of the overall variation (Table 5, Figs 4–8). This variation reflects the adaptive capacity of vegetation to environmental constraints (Fyllas *et al.*, 2009; Meng *et al.*, 2009; Ordoñez *et al.*, 2009; Albert *et al.*, 2010b; Poorter *et al.*, 2010) and it highlights the need for refined plant functional classifications for Earth system modeling. The current approach to vegetation modelling, using few basic PFTs and one single fixed parameter value per PFT (even if this value equals the global or regional mean) does not account for the rather wide range of observed values for related traits and thus does not account for the adaptive capacity of vegetation. A more empirically based representation of functional diversity is expected to contribute to an improved prediction of biome boundary shifts in a changing environment.

There are new approaches in Earth system modelling to better account for the observed variability: suggesting more detailed PFTs, modelling variability within PFTs or replacing PFTs by continuous trait spectra. In the context of this analysis we focused on a basic set of PFTs. This schema is not immutable and there is not one given functional classification scheme. In fact, PFTs are very much chosen and defined along specific needs – and the availability of information. For example, the PFTs used in an individual based forest simulator (e.g. Chave, 1999), are by necessity very different from those used for DGVMs. The TRY dataset will be as important for allowing the definition of new, more detailed PFTs as for parameterizing the existing ones. Some recent models represent trait ranges as state variables along environmental gradients rather than as fixed parameter values. The O-CN model (Zaehle & Friend, 2010) is an example towards such a new generation of vegetation models, also the NCIM model (Esser *et al.*, 2011), or in combination with an optimality approach the VOM model (Schymanski *et al.*, 2009). Finally, functional diversity may be represented by model ensemble runs with continuous trait spectra and without PFT classification (Kleidon *et al.*, 2009). However, compared with current vegetation models, these new approaches will be more flexible with respect to the adaptive capacity of vegetation. The TRY database is expected to contribute to these developments, which will provide a more realistic, empirically grounded representation of plants and ecosystems in Earth system models.

### A unified database of plant traits in the context of global biogeography

The analyses presented here are only a first step to introduce the TRY dataset. To better understand, separate, and quantify the different contributions to trait variation observed in TRY, more comprehensive analyses could be carried out, e.g. variance partitioning accounting for phylogeny and disentangling functional and regional influences or analysis of (co-)variance of plant traits along environmental gradients. An integrative exploration of ecological and biogeographical information in TRY is expected to substantially benefit from progress in the science of machine learning and pattern recognition (Mjolsness & DeCoste, 2001). In principle, we are confronted with a similar challenge that genomics faced after large-scale DNA sequencing techniques had become available. Instead of thousands of sequences, our target is feature extraction and novelty detection in thousands of plant traits and ancillary information. Nonlinear relations among items and the treatment of redundancies in trait space have to be addressed. Nonlinear dimensionality reduction (Lee & Verleysen, 2007) may shed light on the inherent structures of data compiled in TRY. Empirical inference of this kind is expected to stimulate and strengthen hypothesis-driven research (Golub, 2010; Weinberg, 2010) towards a unified ecological assessment of plant traits and their role for the functioning of the terrestrial biosphere.

The representation of trait observations in a spatial or climate context in the TRY database is limited (Figs 2 and 3). This situation can be overcome using complementary data streams: trait information can be spatially expanded with comprehensive compilations of species occurrence data, e.g. from GBIF or herbarium sources. For SLA and leaf nitrogen content we provide an example for combining trait information with species occurrence data from the GBIF database and with climate reconstruction data derived from the CRU database (Fig. 3). Given that the major fraction of variation is between species, the variation of species mean trait values may be used – but with caution – as a proxy for trait variation, as has already been performed in recent studies at regional and continental scales (Swenson & Enquist, 2007; Swenson & Weiser, 2010). Ollinger *et al.* (2008) derived regional maps of leaf nitrogen content and maximum photosynthesis from trait information in combination with eddy covariance fluxes and remote sensing data. Based on these approaches and advanced spatial interpolation techniques (Shekhar *et al.*, 2004), a unified global database of plant traits may permit spatial mapping of key plant traits at a global scale (Reich, 2005).

The relationship between plant traits (organism-level) and ecosystem or land surface functional properties is crucial. Recent studies have built upon the eddy covariance network globally organized as FLUXNET (a network of regional networks coordinating observations from micrometeorological tower sites: http://www.fluxnet.ornl.gov) and inferred site specific ecosystem-level properties from the covariation of meteorological drivers and ecosystem-atmosphere exchange of CO_2_ and water (Baldocchi, 2008). These include inherent water-use efficiency (Reichstein *et al.*, 2007; Beer *et al.*, 2009), maximum canopy photosynthetic capacity (Ollinger *et al.*, 2008), radiation use efficiency and light response curve parameters (Lasslop *et al.*, 2010). How species traits relate to these ecosystem-level characteristics has not been investigated, but should be possible via a combined analysis of FLUXNET and TRY data. For example, it is possible to test the hypothesized correlation between SLA, P, and N content of dominant species with radiation use efficiency and inherent water-use efficiency at the ecosystem level (as implicit in Ollinger *et al.*, 2008). Similarly, patterns of spatially interpolated global fields of biosphere–atmosphere exchange (Beer *et al.*, 2010; Jung *et al.*, 2010a) may be related to spatialized plant traits in order to detect a biotic imprint on the global carbon and water cycles. Such increased synthetic understanding of variation in plant traits is expected to support the development of a new generation of vegetation models with a better representation of vegetation structure and functional variation (Lavorel *et al.*, 2008; Violle & Jiang, 2009).

## Conclusions and perspectives

The TRY database provides unprecedented coverage of information on plant traits and will be a permanent communal repository of plant trait data. The first analyses presented here confirm two basic tenets of plant comparative ecology and vegetation modelling at global scale: (1) the aggregation of trait data at the species level represents the major fraction of variation and (2) PFTs cover a relevant fraction of trait variation to represent functional diversity in the context of vegetation modelling. Nevertheless, at the same time these results reveal for several traits surprisingly high variation within species, as well as within PFTs – a finding which poses a challenge to large-scale biogeography and vegetation modelling. In combination with improved (geo)-statistical methods and complementary data streams, the TRY database is expected to support a paradigm shift in ecology from being based on species to a focus on traits and trait syndromes. It also offers new opportunities for research in evolutionary biology, biogeography, and ecology. Finally, it allows the detection of the biotic imprint on global carbon and water cycles, and fosters a more realistic, empirically grounded representation of plants and ecosystems in Earth system models.

## Supporting Information

**Figure S1.** ‘Funnel graph’ indicating the dependence of standard deviation on sampling size.

**Figure S2.** Outliers identified in case of *SLA* (2404 outliers out of 48 140 entries, after exclusion of duplicates).

**Appendix S1.** Detection of outliers.

**Appendix S2.** Reasoning and consequences of normal distribution on logarithmic scale.

**Appendix S3.** Ranges of plant traits as a function of trait dimensionality.

**Appendix S4.** Reduction of number of species with complete data coverage with increasing number of traits.

**Appendix S5.** Latitudinal range of SLA.
