# Supplementary figures and images for: TRY – a global database of plant traits
Source: Glob Chang Biol. 2011 Sep;17(9):2905–35. doi: 10.1111/j.1365-2486.2011.02451.x (PMC3627314; doi:10.1111/j.1365-2486.2011.02451.x)

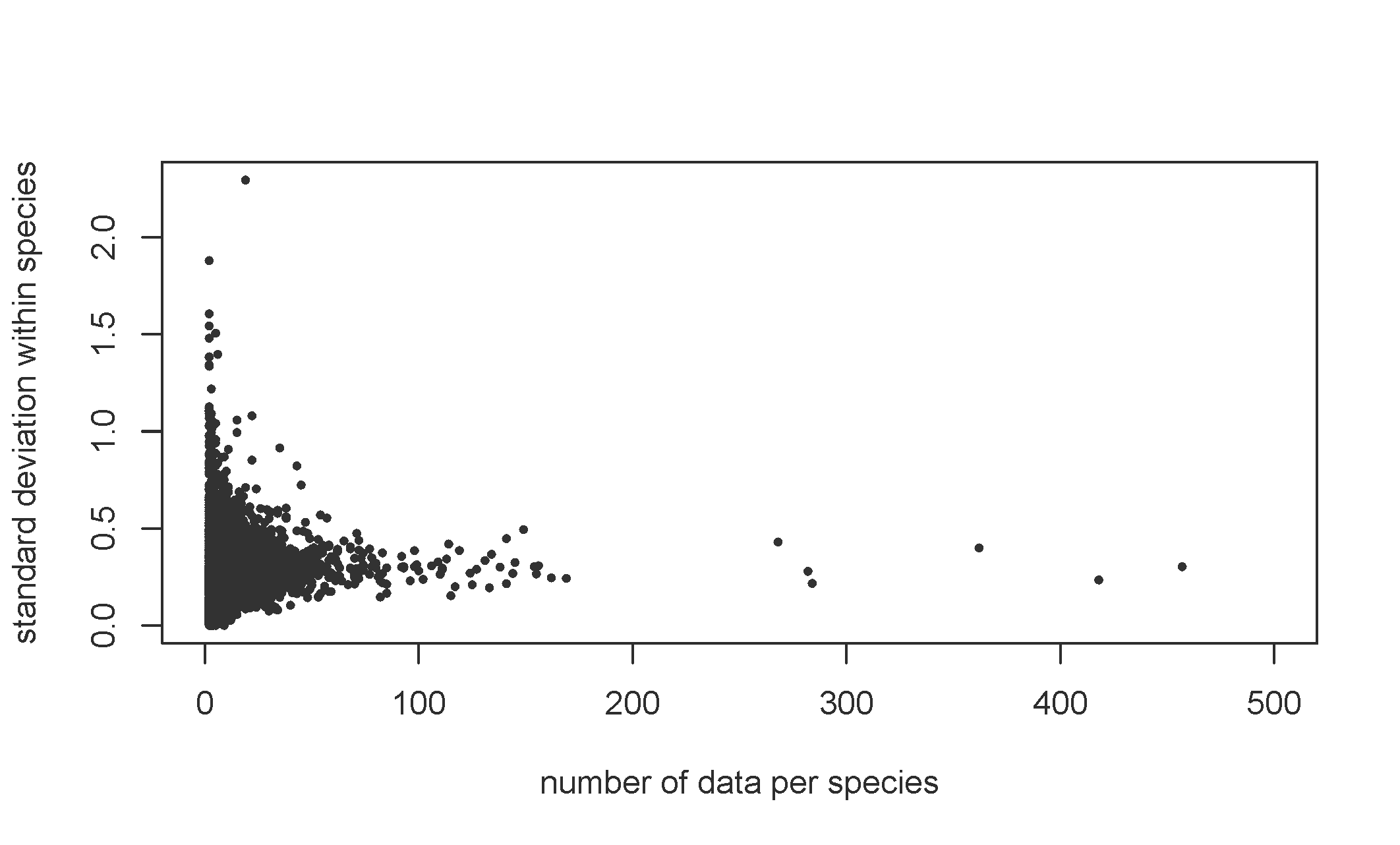

Supplement: Supplementary file 1 [file gcb0017-2905-SD1.tiff]

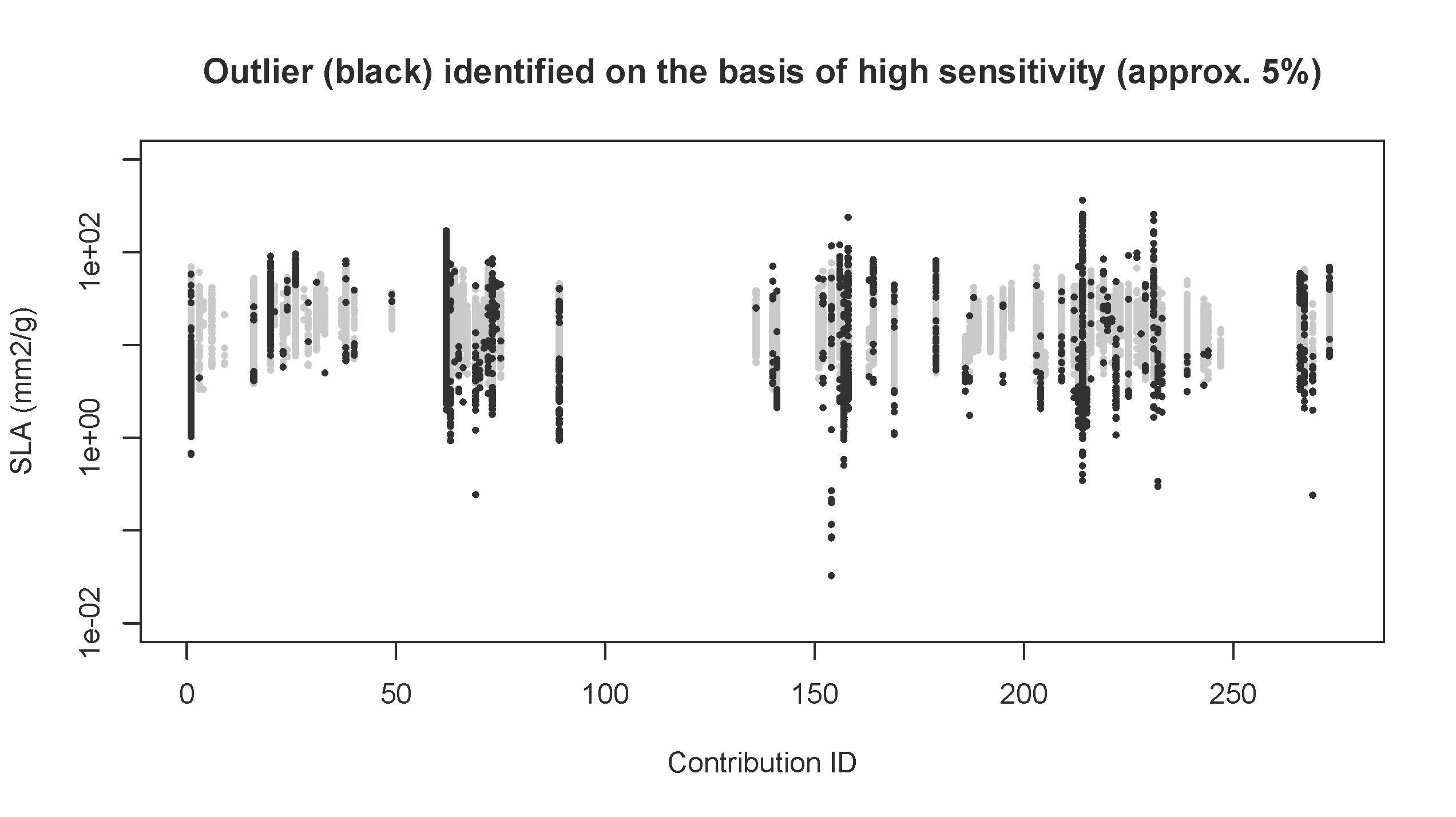

Supplement: Supplementary file 2 [file gcb0017-2905-SD2.tiff]

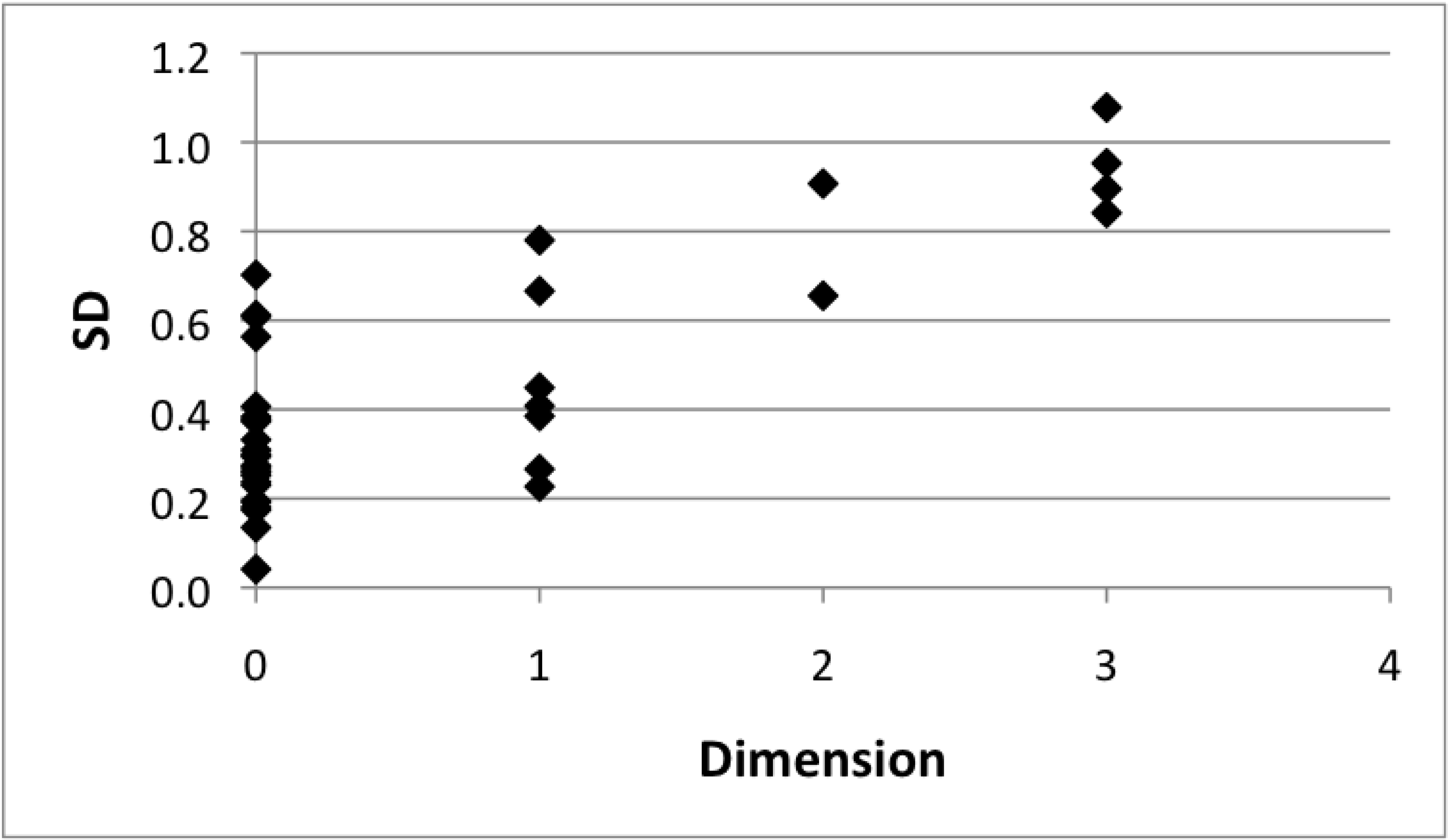

Supplement: Supplementary file 3 [file gcb0017-2905-SD3.tiff]

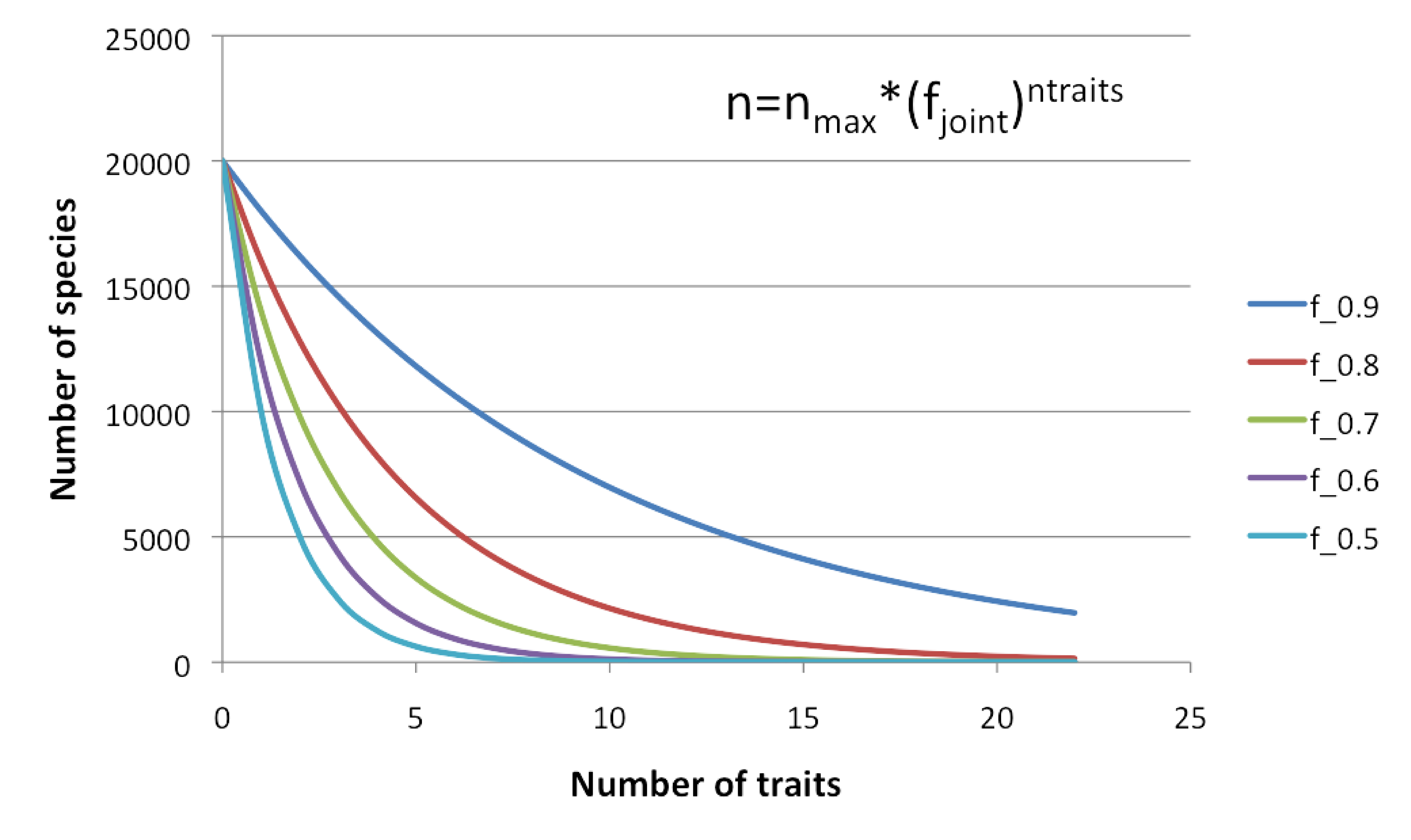

Supplement: Supplementary file 4 [file gcb0017-2905-SD4.tiff]
