# Supplementary material for: TRY – a global database of plant traits
Source: Glob Chang Biol. 2011 Sep;17(9):2905–35. doi: 10.1111/j.1365-2486.2011.02451.x (PMC3627314; doi:10.1111/j.1365-2486.2011.02451.x)
Supplement: Supplementary file 6 [file gcb0017-2905-SD6.doc]

**Supplementary material:**

**S1: Detection of outliers**

We consider individual trait values as outliers if they are not contained within an “accepted range” of values, defined as:

$AcceptedRange=\overline{\ln(x_{i})}\pm a*\left(\overline{SD(\ln(x_{j}))}+b*SD_{SD(\ln(x_{j})}\right)$

i= individual grouping element (respective species, genus, family, PFT)

j= grouping level (e.g. species, genus, family, PFT, or all data)

a, b: factors scaling the sensitivity of outlier detection

x: value of respective trait

The accepted range of values is defined by the mean value of the given group (e.g. a specific species) +/- the mean standard deviation of this grouping level. The mean standard deviation is modified to account for its uncertainty (see Figure S1). The distance of the trait entry under scrutiny from the group mean is calculated in terms of standard deviations. This information is added to the trait information. If the trait entry is out of range for one grouping level, it is marked as an outlier. The accepted range can be formulated with respect to different scaling of the data (e.g. on the original scale or logarithmic scale). Here the approach is formulated on a logarithmic scale, as according to the Jarque-Bera test (Bera and Jarque 1980) most traits were normally distributed on a logarithmic scale; Table 3.

This outlier detection, based on the average standard deviation and its uncertainty, allows a robust detection of outliers also for individual groups with few data entries, e.g. species with only two data entries: both entries could be identified as outliers. In the context of the analyses in this manuscript the scaling factors a and b were set to 2 and 1, respectively. This represents an average range of 2 SD plus the uncertainty of SD. Accordingly about 5% of the data has been identified as outliers and excluded from analyses. In the context of this manuscript we used this rather conservative approach, as individual double- and cross-checking of data for measurement and data errors is not possible for all the traits in the database.

Applying the outlier detection on the aggregation levels of species, genus, family, PFT and for all data, most outliers are detected on the species level in the centre of the distribution (e.g. Figure 1 S1 for *SLA*), only comparatively few entries on the high and low end of the distribution are identified as outliers.

**Figure 1 S1: ‘**Funnel graph’ indicating the dependence of standard deviation on sampling size.

**Figure 2 S1:** Outliers identified in case of *SLA* (2404 outliers out of 48140 entries, after exclusion of duplicates)

**S2: Reasoning and consequences of normal distribution on logarithmic scale**

Our results show that plant traits are typically normally distributed on a logarithmic scale (Table 3). This is most probably due to the fact that they often have a lower bound at zero but no upper bound relevant for the data distribution. Being log-normal distributed has several implications for data analysis and the presentation of results. (1) The standard deviation expressed on a logarithmic scale allows a direct comparison of variation between different traits independent of units and mean values (e.g. table 4 and 5). Providing mean value +/- standard deviation on a logarithmic scale corresponds to a multiplicative relation on the original scale, which corrects for the value of the mean, but also produces an asymmetric distribution, with a small range below and large range above the mean value: e.g. a standard deviation of 0.05 on log-10 scale corresponds to -10.9% and +12.2% on the original scale, while a standard deviation of 1.0 on log-10 scale corresponds to -90% (/10) and +900% (*10) on the original scale. (2) It implies that on the original scale relationships are to be expected being multiplicative rather than additive (Kerkhoff and Enquist 2009). The increase of a trait value by 100% corresponds to a reduction by 50% and not to a reduction by 100%, e.g. a doubling of seed mass from 4mg to 8mg corresponds to a reduction from 4mg to 2mg and not to a reduction from 4mg to 0mg. (3) Log- or log-log scaled plots are not sophisticated techniques to hide huge variations, but the appropriate presentation of the observed distributions (Wright et al. 2004). On the original scale bivariate plots show a heteroscedastic distribution e.g. (Kattge et al. 2009). (4) In the context of sensitivity analysis of model parameters and data-assimilation the trait related parameters and state-variables have to be assumed normally distributed on a logarithmic scale as well (Knorr and Kattge 2005) (e.g. Fig 7 and 8).

**S3: Ranges of plant traits as a function of trait dimensionality**

**S3:** Ranges of plant traits, presented as standard deviation on log-scale, for 50 different traits with respect to dimension. Dimension 1: e.g. length; 2: e.g. area; 3: e.g. volume, mass (variable due to potential changes in three dimensions); all traits that are calculated as fractional values (e.g. mg/g, or g/m2) are attributed a dimension of zero.

**S4: Reduction of number of species with complete data coverage with increasing number of traits**

**S4:** Reduction of number of species with complete data coverage with increasing number of traits. n: number of species with full coverage; n_max_: number of species in case of trait with highest coverage; f_joint_: average fraction of species overlap of different traits; ntraits: number of traits of interest in the multivariate analysis. First experiences show that in TRY f_joint_ is in a range of about 0.5 to 0.7, depending on the selected traits.

**S5: Latitudinal range of SLA**

**S5:** Worldwide range in *SLA* along a latitudinal gradient for the main plant functional types. Density distributions presented as box and whisker plots. The height of boxes indicates the number of respective data entries.
